# Polarization-Dependent Optical Properties and Optoelectronic Devices of 2D Materials

**DOI:** 10.34133/2020/5464258

**Published:** 2020-08-29

**Authors:** Ziwei Li, Boyi Xu, Delang Liang, Anlian Pan

**Affiliations:** Key Laboratory for Micro-Nano Physics and Technology of Hunan Province, College of Materials and Engineering, Hunan University, Changsha, Hunan 410082, China

## Abstract

The development of optoelectronic devices requires breakthroughs in new material systems and novel device mechanisms, and the demand recently changes from the detection of signal intensity and responsivity to the exploration of sensitivity of polarized state information. Two-dimensional (2D) materials are a rich family exhibiting diverse physical and electronic properties for polarization device applications, including anisotropic materials, valleytronic materials, and other hybrid heterostructures. In this review, we first review the polarized-light-dependent physical mechanism in 2D materials, then present detailed descriptions in optical and optoelectronic properties, involving Raman shift, optical absorption, and light emission and functional optoelectronic devices. Finally, a comment is made on future developments and challenges. The plethora of 2D materials and their heterostructures offers the promise of polarization-dependent scientific discovery and optoelectronic device application.

## 1. Introduction

Two-dimensional (2D) materials, as emerging ultrathin material families, exhibit diverse optical, electronic, and magnetic properties, such as flexible energy band design [1–3], anisotropic optical properties [4, 5], spin-valley-coupled physics [6–8], and multifield-tunable light emissions [9, 10], that make them ideal candidates for probing novel scientific problems and exploring potential device applications. These atomic materials show excellent electronic and optoelectronic properties compared to their bulk materials, and they possess natural advantages for system integration in Si-based integrated circuits. With the increasing requirement of device function, the study of optoelectronic properties of 2D materials and devices extends from the traditional detection of optical intensity, photoresponse, and frequency [11–13], towards the harvest or the manipulation of polarized-state-of-light and spin-state-of-particle, such as valley-polarization photoluminescence [14, 15], anisotropic photoresponses [16, 17], and spin-valleytronic devices [18, 19]. The polarized-light-dependent optical properties of 2D materials are worthy to be investigated, which help to reveal the intrinsic physics of strong light-matter interactions, and may contribute to designing smart optoelectronic devices in the field of nanotechnology [20–22].

Since the successful fabrication of semimetallic graphene in 2004 [23], the investigations of 2D materials have attracted great attention. Varieties of 2D semiconductors have been synthesized [24–26], including group-IV monochalcogenides (MX) [27, 28], transition metal dichalcogenides (MX_2_) [25, 29–33], group-IVB trichalcogenides (MX_3_) [34, 35], black phosphorus (BP) [36], and perovskites [37, 38]. With the rapid development of 2D materials, the research interest of 2D science is gradually broadened and focused, especially in polarization-dependent optical properties and devices [39, 40]. In early researches, graphene has been reported to possess linear electronic dispersion at Dirac points, and its band gap can be opened up by interacting with a magnetic field or building a bilayer [41, 42]. Intriguingly, the opened finite band gap can induce two inequivalent valleys at two Dirac points, which leads to the growing interest in 2D valleytronic materials [43]. Beyond graphene, the group of transition metal dichalcogenides (TMD) is theoretically predicted and experimentally reported to show valley-selective circular dichroism, which provides a selection rule that materials can couple polarized photons generating spin electrons and holes [40]. The studies of spin-valley coupling and valleytronic devices based on TMD and TMD heterostructures start to be proposed extensively, including valley polarization photoluminescence (PL) [44, 45], valley Hall effect [46–48], and magnetically induced Zeeman splitting [49, 50].

Distinguished from other widely reported 2D semiconductors, 2D BP owns ultrahigh electron mobility and exhibits anisotropic optical properties along two directions, the armchair (AM) and zigzag (ZZ) directions [51, 52]. The lattice constants of BP along the two perpendicular directions are 3.30 Å and 4.53 Å, respectively, which create the anisotropic energy band and give rise to polarization-dependent optical properties. However, the stabilization of BP remains a great challenge for material synthesis and device fabrication. Much effort has been devoted to design and explore stable anisotropic semiconductors for device applications [53–55]. Some anisotropic materials are brought to our attention, such as ReS_2_ [56], ReSe_2_ [57], TiS_3_ [34], and GaTe [58]. Their structure symmetries and periodically varied Raman shifts have been systematically investigated.

Besides the unique optical properties arising from pristine physics, 2D materials show great merits in flexible heterointegration with nanophotonic structures [59]. Nanophotonic structures, such as plasmonic nanostructures [60–62], metallic nanowires [63, 64], photonic crystals [19, 65], and metasurfaces [66], can interact with light-induced electromagnetic modes or dispersion modulations, which also help to harvest polarized photons making novel optoelectronic devices. Plasmonic nanostructures provide a large amount of free electrons, which can be excited by far-field light, generating near-field electromagnetic enhancements to tailor the behaviors of 2D exciton emissions. Besides, photonic crystals are usually fabricated with periodical dielectric materials, which can suppress the loss and offer high-quality photon modes [67]. The strong coupling between excitons and photons has been extensively investigated in the system of photonic crystal-coupled 2D materials. Metasurfaces are periodic artificial nanostructures, which can be designed to couple 2D semiconductors to change the exciton properties of propagation direction, polarization state, and nonlinear optical response [68–72].

The aim of this review is to summarize polarization-dependent optical properties of 2D materials for the application of next-generation photonic and optoelectronic devices. We firstly illustrate the physical mechanism of polarization-dependent light-matter interactions based on 2D materials. Then, we review the optical properties among polarized-light-dependent Raman shift, PL emission, and light absorption in anisotropic 2D materials. Besides, spin-valley physics and multifield-tunable valley-dependent circular dichroism of TMD have been discussed. In other hybrid structures based on 2D materials, the advanced photonic devices have been proposed among photon emissions and propagations. Furthermore, we also discuss the potential applications of these materials in functional optoelectronic devices. Finally, we draw the outlook of 2D materials and categorize our visions in future research fields for more nanotechnology applications.

## 2. Polarization Mechanism

The polarization-dependent optical properties of 2D materials are arising from the physical nature of materials and heterostructures [73]. It can be concluded as three aspects: anisotropic structure-induced anisotropic energy bands [74, 75], inequivalent valleys in isotropic TMD [40], and the interactions between excitons and polarized plasmons or photons [76]. To discuss the polarization mechanism in the mid- and far-infrared regime, the permittivity and optical conductivity of 2D materials and nanophotonic structures can be described using the semiclassical Drude model [77, 78]. In a simplified model, the polarization contribution of materials in the *z* direction is considered to be homogeneous, and the equivalent relative permittivity (*ε*_*jj*_) and optical conductivity (*σ*_*jj*_) of 2D materials in the *x*‐*y* plane can be described as

(1) $$\begin{array}{c} \varepsilon_{jj}=\varepsilon_{r}+\frac{i\sigma_{jj}}{\varepsilon_{0}\omega t_{2\text{D}}},\;j=x,y, \end{array}$$

(2) $$\begin{array}{c} \sigma_{jj}=\left[\begin{array}{cc} \sigma_{xx} & \sigma_{xy}\\ \sigma_{yx} & \sigma_{yy} \end{array}\right], \end{array}$$

where *ε*_*r*_ and *ε*_0_ are the relative permittivity of 2D materials and vacuum permittivity, respectively. *ω* is the optical frequency. *t*_2D_ is the thickness of 2D materials. The polarization-dependent optical and optoelectronic properties are determined by these parameters.

**Figure 1:**
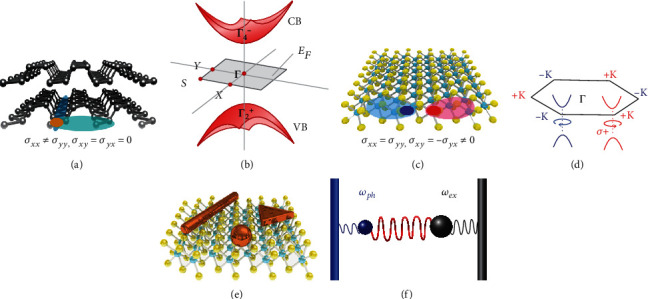
Physical mechanism of polarization-dependent optical phenomena in 2D materials. (a) Anisotropic 2D materials and the schematic view of 3D energy band. (b) TMD materials and their inequivalent valleys in the Brillouin zone. (c) Heterostructures of 2D materials and nanophotonic structures. The schematic view of multibody coupling among excitons, plasmons, and photons.

For anisotropic 2D materials in Figure 1(a), it has been predicted that the effective mass of carriers is highly anisotropic in real space due to the anisotropic atom arrangements, which gives rise to the anisotropic energy band in the *k*-space [79, 80]. First-principle calculations indicate that the polarization-dependent optical responses are dominated by the dipole selection rules based on the symmetry of wave functions in valence and conduction bands [81]. The optical conductivity of 2D anisotropic material exhibits *σ*_*xx*_ ≠ *σ*_*yy*_ and *σ*_*xy*_ = *σ*_*yx*_ = 0, which implies that the optical responses along two directions are different [82]. As an example, Figure 1(b) shows the anisotropic band structure of pristine BP, where the valence band and conduction band are located at the zone center of the *Γ*_2_^+^ and *Γ*_4_^−^ states, respectively.

While for gapped Dirac materials (for example, TMD) in Figure 1(c), the effective mass of carriers is isotropic, but the electronic wave functions in two Dirac valleys “twist” differently. The optical conductivity of 2D TMD can be depicted as *σ*_*xx*_ = *σ*_*yy*_ and *σ*_*xy*_ = −*σ*_*yx*_ ≠ 0. In the schematic view of the Brillouin zone of the MoS_2_ monolayer, two inequivalent valleys (-K and +K valleys) appeared in the neighboring valleys stemming from the characteristic crystal features of TMD materials, involving C_3_ symmetry, inequivalent A-B sublattices, and direct band gap, as shown in Figure 1(d). The valley-selective circular dichroism of the TMD exciton arises from the inverse Berry curvatures in two inequivalent valleys [83].

In particular, graphene is a Dirac semimetal material with linear electronic dispersion, and its optical conductivity tensor is isotropic, depicted as *σ*_*xx*_ = *σ*_*yy*_ and *σ*_*xy*_ = *σ*_*yx*_ = 0. Above all, the optical conductivity of 2D materials can be concluded as

(3) $$\begin{array}{c} \left\{\begin{array}{c} \text{Isotropic}:\sigma_{xx}=\sigma_{yy},\sigma_{xy}=\sigma_{yx}=0,\\ \text{Anisotropic}:\sigma_{xx}\neq \sigma_{yy},\sigma_{xy}=\sigma_{yx}=0,\\ \text{Gapped Dirac}:\sigma_{xx}=\sigma_{yy},\sigma_{xy}=-\sigma_{yx}\neq 0. \end{array}\right. \end{array}$$

For the hybrid systems of nanophotonic structures and 2D materials in Figure 1(e), the situations are more complicated, and they need detailed discussions in each case. In the situation of the weak coupling between 2D materials and nanostructures, the multilayer structure can be simply considered as an effective homogeneous medium, and the effective permittivity *ε*_*jj*_^eff^of hybrid structure along the *x*, *y*, and *z* directions can be described as

(4) $$\begin{array}{c} \varepsilon_{jj}^{\text{eff}}=\frac{\varepsilon_{jj}t_{2\text{D}}+\varepsilon_{c}t_{c}}{t_{2\text{D}}+t_{c}},\;j=x,y, \end{array}$$

(5) $$\begin{array}{c} \varepsilon_{jj}^{\text{eff}}=\frac{\varepsilon_{jj}\varepsilon_{c}\left(t_{2\text{D}}+t_{c}\right)}{t_{2\text{D}}\varepsilon_{c}+t_{c}\varepsilon_{2\text{D}}},\;j=z, \end{array}$$

where *t*_2D_ and *t*_*d*_ are the thicknesses of 2D materials and coupled structures, respectively. *ε*_*c*_ is the permittivity of the coupled structure. The coupled structures should be dielectric materials, plasmonic nanostructures, or others. In the visible spectral range, the physics of multibody coupling between excitons in 2D materials and plasmons/photons in coupled structures can be described in harmonic-oscillator models. In Figure 1(f), the blue and black balls are considered as oscillators possessing resonance frequencies of *ω*_*ph*_ and *ω*_*ex*_, respectively. The resonance frequencies of optical absorption and photon emission can be detuned by each oscillator. The above functions describe the physical mechanism of polarization-dependent optical properties of 2D materials.

## 3. Anisotropic Materials and Optical Properties

Different kinds of anisotropic 2D materials have been reported widely, among BP, group-IV monochalcogenides (MX, M=Sn, Ge, Re, X=S, and Se), group-IVB trichalcogenides (MX_3_, M=Ti, Zr, X=S, and Se), and others. The anisotropic optical properties arise from low-symmetric crystal structures, which usually can be observed in orthorhombic, monoclinic, and triclinic structures. Here, we review the anisotropic atom structure, polarization-dependent Raman shift, light absorption, and PL spectra of typical anisotropic 2D materials.

### 3.1. Anisotropic Structure and Raman Shift

**Figure 2:**
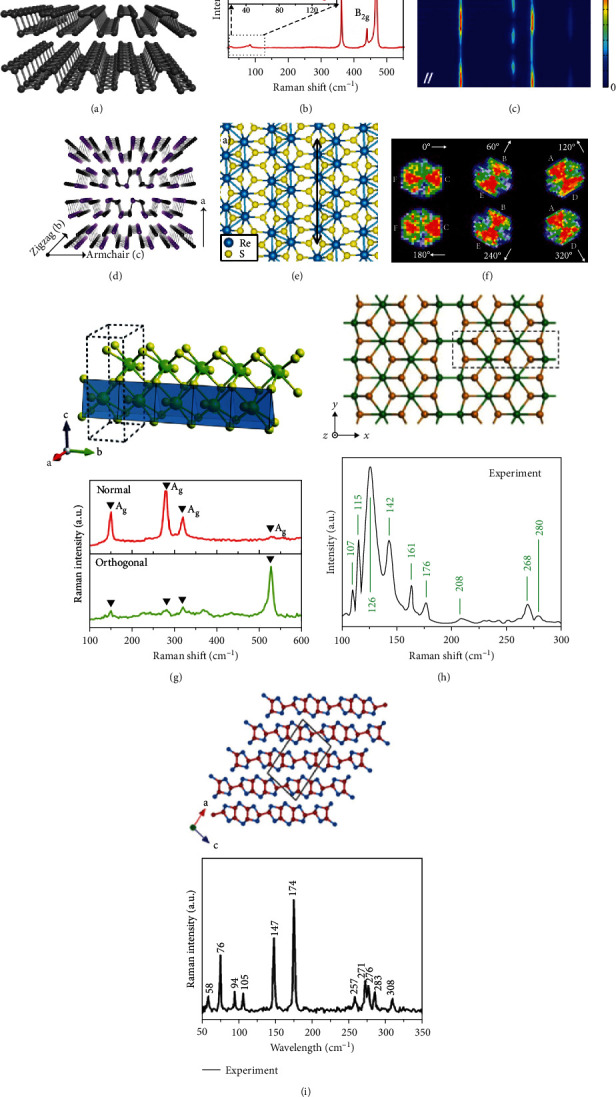
Anisotropic 2D materials and polarization-dependent Raman spectra. (a) Atomic structure of BP with puckered honeycomb configuration. (b) Characteristic Raman peaks of BP at high-frequency modes. Inset shows the B modes of BP at low wavenumbers. (c) Intensity map of periodic Raman signals of BP. (d) Atomic structure of few-layer GeSe with puckered honeycomb configuration. (e) Distorted 1T structure of ReS_2_ flake. (f) ANRS mapping data at 214 cm^−1^ peak with different polarization angles. (g) Atomic structure of ZrS_3_ flakes. The dotted black line represents the primitive monoclinic cell. The Raman peaks of ZrS_3_ obtained along the *b*-axis (red plot) and *a*-axis (green plot). (h) Top view of GaTe monolayer. Experimental Raman spectrum of thick GaTe flakes at room temperature. (i) Crystal structure and Raman signals of layered GaAs flakes. Panel (a) is reproduced with permission from Ref. [86], copyright 2020 *Advanced Materials*. Panel (b) is reproduced with permission from Ref. [90], copyright 2015 *Nano Letters*. Panel (c) is reproduced with permission from Ref. [92], copyright 2015 *ACS Nano*. Panel (d) is reproduced with permission from Ref. [99], copyright 2017 *Journal of the American Chemical Society*. Panel (e) is reproduced with permission from Ref. [104], copyright 2015 *Nano Letters*. Panel (f) is reproduced with permission from Ref. [105], copyright 2016 *Nano Letters*. Panel (g) is reproduced with permission from Ref. [35], copyright 2016 *Nanoscale*. Panel (h) is reproduced with permission from Ref. [108], copyright 2016 *ACS Nano*. Panel (i) is reproduced with permission from Ref. [96], copyright 2018 *Advanced Functional Materials*.

Bulk BP owns an orthorhombic crystal structure with point group D_2h_^18^ and space group *Cmce*. As the layer number decreases, the point group and space group of odd-layer BP film appears to be D_2h_^7^ and *Pmna*. But the even-layer BP belongs to space group *Pmca* and point group D_2h_^11^ [84, 85].  Figure 2(a) shows the atomic structure of BP, where each atom is covalently bonded with three adjacent phosphorus atoms forming a puckered honeycomb structure. There are two in-plane directions according to periodic atom arrangements: one is the armchair direction along the *x* direction and the other is the zigzag direction along the *y* direction [86]. From DFT calculations, the electronic band structures of BP are varied with the number of layers but always keep a regime of direct band gap. As the layer number decreases, the optical band gap changes from 0.3 eV (bulk) to 2 eV (monolayer) [87, 88].

Raman spectroscopy provides a direct, rapid, and sensitive characterization method to record the structural and the optical information of materials, which has been widely used to study intralayer lattice vibrations and interlayer coupling modes of BP [89].  Figure 2(b) shows the Raman spectra of few-layer BP at both high-frequency and low-frequency ranges. Three typical high-frequency peaks of A_g_^1^, B_2g_, and A_g_^2^ are observed to be located at 362.3 cm^−1^, 439.2 cm^−1^, and 467.1 cm^−1^, respectively. The out-of-plane breathing modes of few-layer BP at low frequency are determined as 26.2 cm^−1^, 75.6 cm^−1^, and 85.6 cm^−1^, respectively, as shown in the inset of Figure 2(b). The breathing modes are extremely sensitive to the interlayer interaction, and DFT methods are developed to simulate the breathing modes and to identify the layer number of BP (2-8 layers) [90].

Polarized Raman spectroscopy is a powerful and popular method to study the crystal symmetry, where polarization direction of detection can be controlled either parallel or perpendicularly to the polarization direction of incident light [91].  Figure 2(c) exhibits the polarization-dependent Raman spectra of BP under parallel polarization configuration, where the intensity variation period of A_g_^1^ and A_g_^2^ peaks are 180°, while it is 90° for the B_2g_ mode. The maximum intensities of the A_g_^1^ and A_g_^2^ modes are observed at 0° and 180°, which mean excitation and the detection polarization directions are aligned with the armchair direction. For crosspolarization configuration, both of the intensity variation periods of A_g_^1^ and A_g_^2^ peaks are observed to be 90°, and the variation period of B_2g_ keeps 90° the same as the measurements in the crosspolarization configuration. The difference of intensity variation period of A_g_ in two situations can be understood in the analysis of phase difference in Raman tensors [92].

The family of group-IV monochalcogenide-layered materials (MX), such as SnS, SnSe, GeS, and GeSe, has a similar crystal structure of the orthorhombic system as BP. The only difference is that the M atom is covalently bonded with three adjacent X atoms to form the puckered M-X layers [93–98]. As an example, Figure 2(d) shows the atomic structure of few-layer GeSe [99]. Duan et al. successfully exfoliated thin-layered SnSe flakes from a single crystal, and the in-plane anisotropic properties are systematically studied. Four characteristic Raman modes are clearly observed as A_g_^1^ (69.5 cm^−1^), A_g_^2^ (130.6 cm^−1^), A_g_^3^ (149.5 cm^−1^), and B_3g_ (109.0 cm^−1^). The spectral intensity of each Raman mode varies individually as the angle increases [100].

Another group of anisotropic 2D materials is ReX_2_ (X=S, Se), which owns a triclinic crystal exhibiting more unique polarization-dependent Raman features [101, 102]. ReS_2_ has an X-Re-X structure, where the upper X atoms are rotated by 180° with respect to the lower X atoms, forming a Re-centered octahedral.  Figure 2(e) shows the crystal structure of distorted 1T-ReS_2_, where the red line represents the direction of Re chains [103, 104]. The anisotropic structure arises from various bonding states of X atoms; X atoms located inside the narrow Re strings have a shorter Re-X bonding length, but those in wider channels have a longer Re-X bonding length. Angle-resolved Raman mapping of 214 cm^−1^ mode is shown in Figure 2(f), where the neighboring triangle domains are inhomogeneous showing polarization-selective amplification of Raman intensity [105].

Transition metal trichalcogenides (MX_3_, M=Ti, Zr, Hf, X=S, Se, and Te) are typical 2D anisotropic materials, belonging to space group P2_1_/m with a monoclinic crystal structure [106]. In Figure 2(g), TiS_3_ resembles as a chain-like structure along one of the lattice directions, and the unit cell of TiS_3_ contains two titanium and six sulfur atoms. The maximum Raman intensity is detected when the polarization direction of excitation is parallel to the *b*-axis direction [35]. Another work demonstrates some unusual lattice vibrations of TiS_3_ at high-pressure environments. Four prominent Raman modes are determined as “out-of-plane” vibrations [107]. The intensities of A_g_^Inter^ peaks show two- or fourfold periodic features, while other peaks show negligible polarization responses. Interestingly, the Raman shift of the A_g_^S-S^ mode exhibits an unconventional negative pressure dependence, which can be explained in that the reduced interlayer distance at high pressure increases the orbital interaction between adjacent layers.

Figure 2(h) shows the crystal structure of GaTe with a low symmetry of the C_2h_^3^ space group. Their unique atom arrangements give rise to in-plane anisotropic properties. In experiments, eight Raman modes can be detected, emerging at 107, 115, 126, 142, 161, 208, 268, and 280 cm^−1^. Five of them are A_g_ modes (107, 115, 208, 268, and 280 cm^−1^), one is the B_g_ mode (161 cm^−1^), and the other two are double-resonant modes (126 and 142 cm^−1^) [108]. Similar to GaTe, 2D GeAs possesses sensitive responses to polarized excitations, as shown in Figure 2(i). Eleven Raman active modes are detected in the parallel configuration, including eight A_g_ modes (94, 105, 147, 174, 271, 276, 283, and 308 cm^−1^) and three B_g_ modes (58, 76, and 257 cm^−1^). However, seven Raman active modes can be observed under the detection of cross configuration, including three A_g_ modes (147, 174, and 276 cm^−1^) and four B_g_ modes (76, 86, 243, and 257 cm^−1^). Besides, the anisotropic Raman signals are sensitive with excitation energy, phonon energy, and flake thickness [96, 109].

### 3.2. Anisotropic Optical Absorption

**Figure 3:**
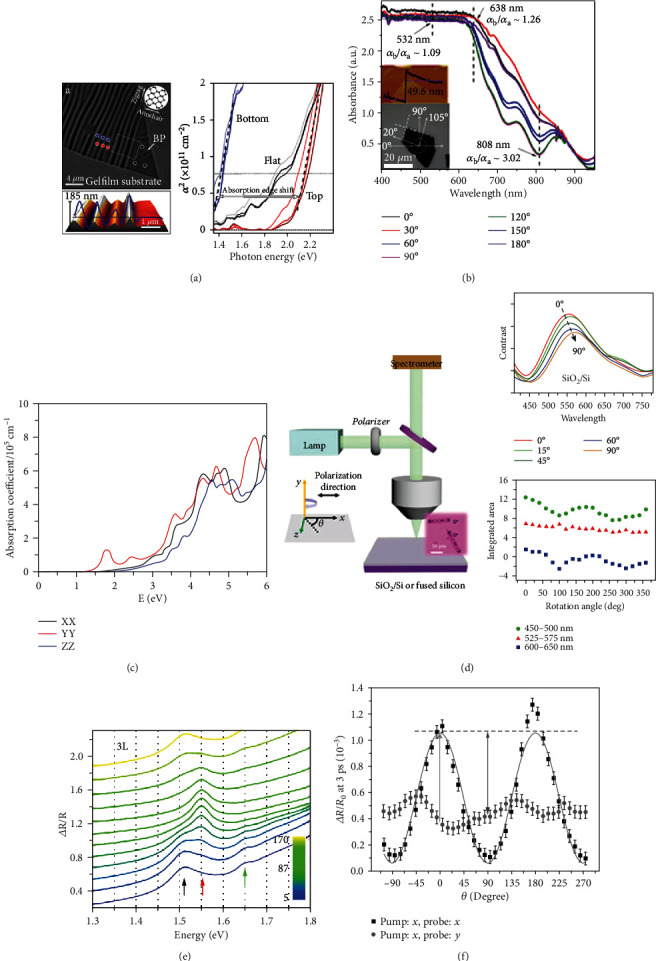
Polarization-dependent light absorption of anisotropic 2D materials. (a) SEM image and absorption spectra of rippled BP film. (b) Polarization-resolved absorption spectra of GeSe in the visible spectral range. Inset shows SEM and AFM images. (c) Calculations of optical absorption coefficients of TiS_3_ monolayer along different polarization directions. (d) Polarization-dependent reflectance spectra map for monolayer 1T′-MoTe_2_ flake on SiO_2_/Si substrate. (e) Polarization-resolved reflection contrast spectra of 3L ReS_2_. (f) Time-resolved differential reflection signals of ReS_2_ detected at *x*- and *y*-polarized directions. Panel (a) is reproduced with permission from Ref. [112], copyright 2016 *Nano Letters*. Panel (b) is reproduced with permission from Ref. [99], copyright 2017 *Journal of the American Chemical Society*. Panel (c) is reproduced with permission from Ref. [106], copyright 2015 *Physical Chemistry Chemical Physics*. Panel (d) is produced with permission from Ref. [122], copyright from 2019 *Small*. Panel (e) is reproduced with permission from Ref. [126], copyright 2016 *ACS Photonics*. Panel (f) is reproduced with permission from Ref. [128], copyright 2015 *Small*.

For ultrathin 2D materials, the detection of optical absorption is mainly obtained from the difference of reflection spectra between substrate and 2D materials [110]. The anisotropic electron-photon and electron-phonon interactions have been investigated in BP flakes [111].  Figure 3(a) presents the SEM image of a strain-engineered BP film with ripples. The absorption peaks can be tailored by adding strain force [112]. Another work shows the absorption spectra of monolayer BP on a sapphire substrate with h-BN capping layer. The layer-dependent absorption spectra have been systematically studied, where more absorption peaks appear to be observed in thick flakes, and their energies shift to low energy as the layer number increases from 1 to 5. The optical bandgaps of the monolayer, bilayer, and trilayer BP are determined to be 1.73 eV, 1.15 eV, and 0.83 eV, respectively [113]. At a critical doping level, the material becomes a Dirac semimetal showing linear dispersion in the armchair direction and quadratic dispersion in the zigzag direction [74]. Besides, the anisotropic optical absorption of BP can be manipulated by strain force. Theoretical studies predict that the band gap of BP decreases when the transverse compression increases, while the anisotropic behaviors are reserved [114, 115].

When light propagates into a material medium, the energy of light decreases as the optical path increases. The optical absorption coefficiency *α* represents the physical meaning that the energy of propagating light will reduce to *α*^−1^ times the original at a certain distance. In the near-infrared spectral range, 2D GeSe nanoflakes show anisotropic properties in optical absorption, and polarized-angle-resolved absorption spectra of GeSe nanoflakes are displayed in Figure 3(b) [99, 116]. The anisotropic absorption ratios at specific wavelengths are marked by dashed lines, and *α*_*y*_/*α*_*x*_ are determined to be 1.09 at 532 nm, 1.26 at 638 nm, and 3.02 at 808 nm, respectively. Another work reports a visualized imaging of GeSe flakes using azimuth-dependent reflectance difference microscopy (ADRDM) and polarization-resolved optical microscopy (PROM). These experimental measurements demonstrate the strong band dispersion anisotropy of GeSe along the *x* and *y* directions [117].

MX_3_ (M=Ti, Zr; X=S, Se, and Te) attracts great attention due to its anisotropic properties of optical absorption. Theoretical calculations demonstrate that these materials are semiconductors with the band gaps ranging from 0.57 to 1.90 eV, and they possess highly anisotropic optical properties due to their structural anisotropy [118, 119]. The variation of the absorption coefficient of monolayer TiS_3_ under different polarizations is show in Figure 3(c), where several peaks around 2.0 eV, 2.4 eV, 3.0 eV, and 3.5 eV appear with anisotropic features [106]. Similarly, monolayer TiSe_3_ shows anisotropic absorption at 1.5 eV, 2.4 eV, and 3.6 eV. It indicates that monolayer TiS_3_ is a promising anisotropic material in the visible spectral range, while monolayer TiSe_3_ is an outstanding candidate for both near-infrared and visible spectra.

WTe_2_ and MoTe_2_ have drawn a refreshing attention due to their robust growth and phase engineering between semiconductor (2H) and semimetal properties (1T′). In particular, they are predicted to own intriguing physics among quantum spin Hall insulators, large magnetoresistance, and superconductivity [120, 121]. Recently, the anisotropic optical absorptions of trilayer 1T′ MoTe_2_ have been reported from CVD-grown samples, as shown in Figure 3(d). The angle-resolved absorption spectra demonstrate that the strong anisotropic absorption is observed in blue (450-500 nm) and green regions (525-575 nm) [122]. Another work presents the contrast difference of reflectance measurements, where the contrast is high at the wavelength below 450 nm. In the range of 450–650 nm, the contrast shows the maximum intensity at zero angle (along the main axis of the flake), and a clear symmetry can be observed exhibiting a repeated period of 180° [123].

ReS_2_ is one of the group-VII transition metal dichalcogenides, which possesses anisotropic exciton absorption due to the reduced crystal symmetry [56, 124, 125].  Figure 3(e) shows the reflection contrast spectra of monolayer ReS_2_, where the dramatic shifts of the absorption peaks arise from the competition of two near-gap excitons (labeled as red and black arrows) [126]. The spectral weight can be plotted in a polar map as a function of light polarization. Both the peak intensities of two excitons show a periodic change of 90° from the maximum to the minimum, but the minimum intensity appears at 5° (95°) for the red (black) one. Moreover, the direct bandgap exciton absorption of 3L ReS_2_ shifts red to low energy, and the property of linearly polarized optical absorption remains the same.

The time-resolved transient absorption measurement provides detailed dynamic descriptions to understand the anisotropic absorption when the light polarization is parallel or perpendicular to the Re atomic chains [127]. First, the pump light is selected as an *x*-polarized state, and the time-resolved differential reflection signals at *x*- and *y*-polarized are compared in two sample orientations (0 and 90°). All the decay processes contain two components: the fast one is the energy relaxation of hot carriers (~10 ps), and the low one is the exciton lifetime (~40 ps). It can be concluded that the exciton dynamics are independent with the probe polarization and sample orientation.  Figure 3(f) shows the probe delay of 3 ps as a function of degrees for different polarized states of the probe. It shows a maximum factor of 2.5 in the anisotropic difference of absorption coefficient [128]. Another work utilizes a linearly polarized laser pulse to control the optical stark shift of two energetically separated exciton states. Benefiting from the light-reduced symmetry and different excitonic transition dipole moments, the anisotropic exciton absorption shows a completely distinct dependence with varying polarized angles [129].

### 3.3. Anisotropic Photoluminescence

**Figure 4:**
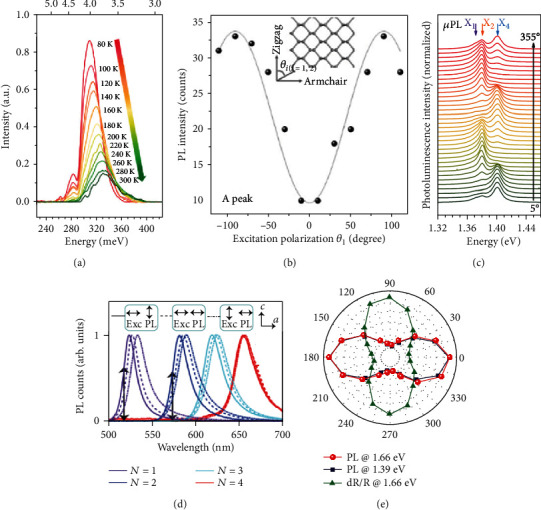
Anisotropic PL spectra of typical anisotropic 2D materials. (a) PL spectra of 46 nm BP at various temperatures. (b) Polarization-dependent PL intensity of monolayer BP on PECVD oxide/Au substrate. (c) Measured normalized PL spectra of 60 nm 1T′-ReSe_2_ as a function of polarization angle. Three characteristic peaks are noted as *X*_1_, *X*_2_, and *X*_4_. (d) Polarization-resolved PL spectra for 2D hybrid perovskite with the layer number ranging from *N* = 1 to *N* = 4. (e) The PL intensities of GaTe flakes at 1.66 eV and 1.39 eV present highly anisotropic features. Panel (a) is reproduced with permission from Ref. [133], copyright 2019 *Nano Letters*. Panel (b) is reproduced with permission from Ref. [134], copyright 2016 *Advanced Materials*. Panel (c) is reproduced with permission from Ref. [136], copyright 2017 *Nano Letters*. Panel (d) is reproduced with permission from Ref. [138], copyright 2018 *Physical Review Letters*. Panel (e) is reproduced with permission from Ref. [58], copyright 2017 *Advanced Materials*.

The low-symmetry and anisotropic screening exciton could lead to the anisotropic PL of 2D materials. As discussed before in BP flakes, the anisotropic light absorption can generate more excitons in specific polarization directions [130]. Besides that, regardless of the polarization direction of excitation, the intensity of PL emission in the *y* direction (zigzag) is always lower than that in the *x* direction (armchair). It has been illustrated in theoretical calculations that the Coulomb interaction is highly anisotropic, which results in the stronger bond of carriers and the weak emission along the *y* direction [131, 132]. Benefitting from the contribution of anisotropic absorption and Coulomb screening, the *x*-polarized PL intensity of BP is quite higher than that of the *y*-polarized state [79]. Polarization-dependent PL spectra of BP with various layer numbers have been investigated. The energies of PL peaks along the *x*-polarization are 1.7 eV, 1.1 eV, and 0.8 eV for 1L, 2L, and 3L BP, respectively. The temperature and thickness-dependent PL spectra demonstrate that the peak energy can be tailored in a wide spectral range, as shown in Figure 4(a) [133]. Furthermore, the emission of the BP exciton can be brightened by modifying the dimensionality of the exciton from quasi-1D to 0D on a PECVD oxide/Au substrate.  Figure 4(b) shows that the luminescence quantum yield has been increased at least 33.6 times, but the anisotropy of the exciton decreases a little, which is stemming from the defect-induced reduction of the local symmetry [134].

The anisotropic exciton in ReX_2_ has been investigated among bulk, few-layer, and monolayer [126]. The energy of the optical band gap exhibits a strong blue shift from 1.37 eV to 1.50 eV as the layer number decreases [135]. Theoretical calculations demonstrate that the anisotropic exciton is highly confined with a large binding energy of 860 meV for the monolayer and 120 meV for the bulk.  Figure 4(c) presents a polarization-resolved microphotoluminescence (*μ*PL) spectroscopy of 60 nm ReSe_2_ flakes at 10 K, where the variation tendency of *X*_1_, *X*_2_, and *X*_4_ excitons can be clearly observed at various polarization angles, indicating that these excitons are strongly polarized along different crystal directions [136].

2D perovskites are rising star semiconductors with large light absorption and photoelectric conversion efficiency. In recent reports, by designing alternating layers of inorganic and organic sublattices, the anisotropic or chiral excitons have been reported widely [38, 137]. Interestingly, the exciton with negative relative permittivity exhibits a hyperbolic dispersion, which has been realized in 2D perovskites prepared by dielectric-coating techniques. By controlling the polarization direction of excitation and detection parallel or perpendicularly, the center of the PL peak shifts obviously for low members (*N* = 1, 2), which arises from the large anisotropy in *k*-space and the Stokes shift between the absorption and emission spectra, as shown in Figure 4(d) [138]. Besides, by incorporating chiral molecules with perovskite nanocrystals, the anisotropic light absorption and chiral exciton emission have been proposed extensively [93, 139–141].

Recently, experiments demonstrate that low-dimensional TiS_3_ and ZrS_3_ also exhibit anisotropic PL. It has been found that the polarization anisotropy of pseudo-1D ZrS_3_ appear smaller as compared to 1D nanowire materials. Such phenomena can be understood that the small anisotropy difference in effective mass prevents the formation of a linearly polarized exciton [34, 35]. The anisotropic PL is also detected in 2D GaTe and GaAs [142, 143]. The first demonstration of anisotropic PL of vapor-phase-synthesized GaTe is well grown on GaAs (111), Si (111), and c-cut sapphire substrates.  Figure 4(e) shows the angle-resolved PL intensities of *X*_0_ (1.66 eV) and *X*_sub_ (1.39 eV), where the variation trend shows very similar angular-dependence with a twofold symmetry [58]. And it shows similar results for 2D GeS samples; the direct band gap exciton shows a peak at ~1.66 eV exhibiting an anisotropic variation [93].

## 4. Valleytronic Materials and Valley Polarization

The emergence of 2D Dirac materials with hexagonal lattices attracts great interest in the research field of valleytronics, such as gapped graphene and TMD. They possess two inequivalent valleys in K and K′ points in the Brillouin zone, which can couple far-field circularly polarized light-generating spin carriers in materials. The two valleys keep time-reversal symmetry with each other due to the existence of opposite Berry curvatures. Here, we review the 2D valleytronic materials and discuss the excitation and the manipulation of valley carriers, as well as the valley polarization PL.

### 4.1. Dirac Materials and Inequivalent Valleys

The recent research in exploring polarized optical properties of 2D TMD has been focused on the detection of valley polarization, which arises from the manipulation of the valley degree of freedom [144, 145]. Similar to spin electrons owing an internal quantum degree of freedom, the TMD have band structures composed of two inequivalent valley “states,” namely, valley degree of freedom. The ability to control valley degree of freedom arises from the existence of Berry phase-related physical properties and strong spin-orbital coupling [40, 146]. The former is a gauge-independent pseudovector creating the essential difference of two inequivalent valleys, which usually can be considered as an effective magnetic field in the reciprocal space. The latter plays a significant role in inducing the interplay between the spin and the pseudospin.

**Figure 5:**
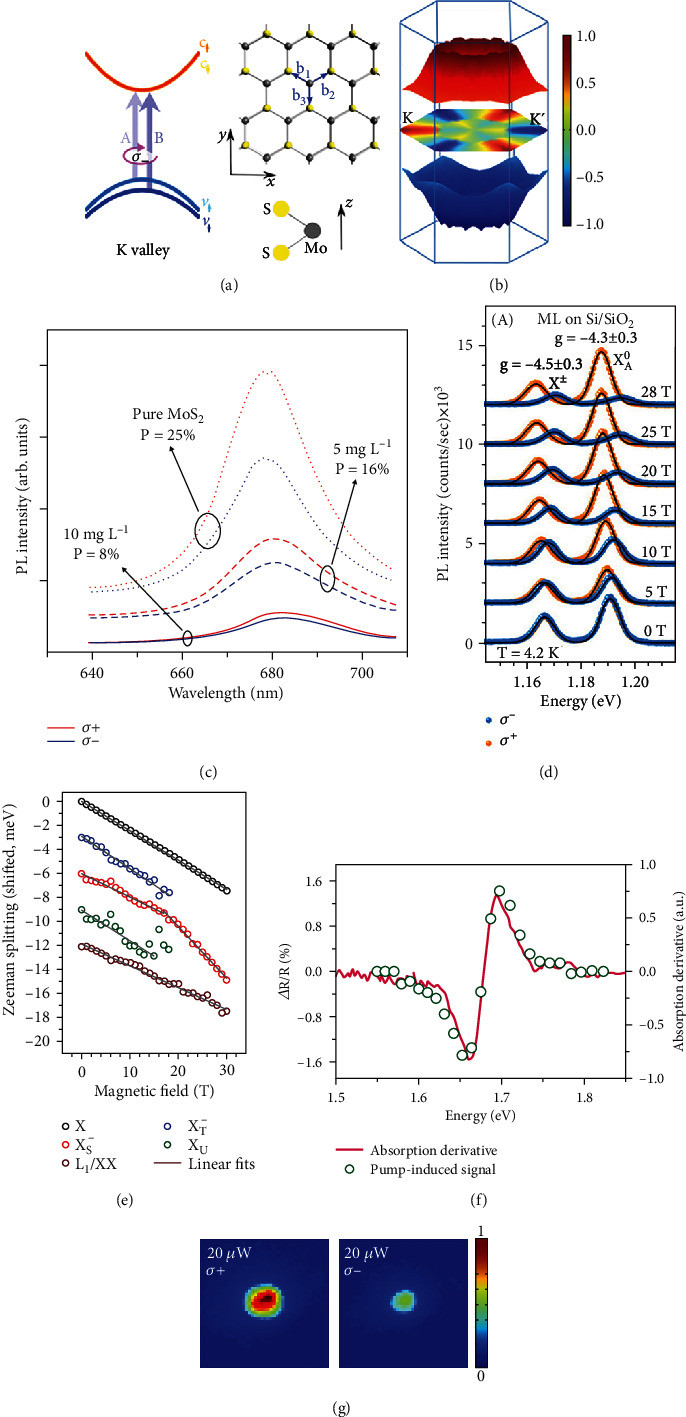
Valley physics and valley polarization PL. (a) Atom structure and valley selection rule of MoS_2_ monolayer. (b) Valley pseudospin and Berry curvature in honeycomb BN monolayer. (c) Valley polarization PL tailored by molecule charge doping. (d) Valley Zeeman effect of monolayer MoTe_2_ at low temperature. (e) The energy of Zeeman splitting of various excitons as a function of the magnetic field. (f) Ultrafast laser-induced pseudomagnetic field for the manipulation of valley exciton in WSe_2_. (g) Spatial PL intensity maps of the *σ*+ (left) and *σ*− (right) interlayer exciton under 20 *μ*W excitation. Panel (a) is reproduced with permission from Ref. [151], copyright 2014 *Physical Review B*. Panel (b) is reproduced with permission from Ref. [152], copyright 2017 *Nano Letters*. Panel (c) is reproduced with permission from Ref. [156], copyright 2015 *Advanced Materials*. Panel (d) is reproduced with permission from Ref. [159], copyright 2016 *Nano Letters*. Panel (e) is reproduced with permission from Ref. [163], copyright 2016 *Nano Letters*. Panel (f) is reproduced with permission from Ref. [164], copyright 2014 *Science*. Panel (g) is reproduced with permission from Ref. [167], copyright 2016 *Science*.

Graphene is well known as a Dirac semimetal, and the Dirac cones in the Brillouin zone provide the platform to generate valley current utilizing its unique edge mode [43], defect line [18], and local strain [147]. Furthermore, inequivalent valleys can be induced and studied by breaking the inversion symmetry or opening the band gap of graphene [41, 42]. However, graphene is not an ideal material to study the valley polarization PL due to its limited ability in opening the band gap. Beyond graphene, the group of TMD is promising as semiconductors for valleytronics, due to their direct band gaps in the visible spectral range [148, 149]. As one of the typical TMD materials, the MoS_2_ monolayer has a hexagonal lattice structure with C3 symmetry. In a crystal unit of the 2H phase, each Mo atom sits in the center of a trigonal prismatic cage formed by six sulfur atoms. The inversion symmetry is broken in this structure regime. In a top view of the MoS_2_ monolayer, the honeycomb lattices are shown with two sets of colors, where yellow and blue balls represent the S and Mo atoms, respectively [150]. In electronic energy bands, the energy extremum points are expected to locate at corners of the hexagonal Brillouin zone (K and K′ points).  Figure 5(a) shows the band structure at the K valley and the atomic structure of MoS_2_ monolayer [151]. Based on the optical transition between the conduction band and the valence band in momentum space, two valleys absorb left- and right-hand circularly polarized light separately [15].

Based on the common features of hexagonal 2D materials, involving C3 symmetry, inequivalent A-B sublattices, and direct band gap at K point, the pseudospin valleys can be explained well in physics. However, few work studies the significant role of the symmetry in creating inequivalent valleys. A recent study discusses the valley polarization of atom-doped 2D hexagonal boron nitride (*h*-BN) [152]. B and N atoms are replaced with carbon atoms in a unit cell at specific locations, and a series of *h*-(BN)*_x_*C_1-*x*_ are calculated using the ab initio calculations. The doped atoms break the crystal period and the symmetry; however, the valley polarization can even survive against the lattice disorder and the symmetry protection. In Figure 5(b), the *k*-resolved degree of optical polarization shows the perfect absorption of circularly polarized photon at K points in the Brillouin zone. This work enriches the family of 2D materials beyond the protection of C3 symmetry.

### 4.2. Manipulation of Valley Exciton

Under the optical pump excitation, valley carriers and excitons carry the quantum state information of polarized photons. The manipulation of quantum information in valley excitons plays an essential role in valleytronics [153–155]. Several methods have been devoted to tailor the valley polarization PL. In hybrid 2D flakes of graphene quantum dot- (GQD-) overlapped MoS_2_, an effective charge transfer is induced from GQDs to the MoS_2_ substrate with reduced dielectric screening, as shown in Figure 5(c) [156]. The PL peak red-shifting, valley PL attenuation can be effectively modulated by controlling the doping process. At a high doping level, the energy level near the bottom of the conduction band degenerates resulting in the spin flip of the electron. The growth of the MoS_2_ monolayer on the GaN substrate was reported with CVD technology [157]. Compared with CVD-grown MoS_2_ on the SiO_2_/Si substrate, the degree of valley polarization is enhanced from 19% to 33% at room temperature. These results are remarkable that such high degrees can be reserved at room temperature, because valley polarization usually vanishes less than 10% at room temperature due to the intravalley scattering. The time-resolved PL spectra provide the dynamic reason that the faster exciton decay rate observed from MoS_2_/GaN could lead to a higher valley polarization degree.

In the regime of broken inversion symmetry, electrons in K and K′ valleys can have finite orbital contribution to their magnetic moment. The magnetic moment at two valleys has equal magnitude but opposite signs, which enables the opposite energy shifts under a magnetic field. The lifting of degeneracy between the two valleys allows the possibility of controlling valley pseudospin via the Zeeman effect [158].  Figure 5(d) shows the helicity-resolved micro-PL spectra of the neutral exciton and the charged exciton (dots) in the MoTe_2_ monolayer as a function of the magnetic field. Both the neutral and charged exciton transitions show clear Zeeman splitting [159]. Srivastava et al. reported the Zeeman effect of the WSe_2_ monolayer under various vertical magnetic fields up to 8.4 T [160]. The energy splitting between two spectra with opposite helicities is clearly observed. The magnetic field creates a sizeable increase in the degree of valley polarization. Xu et al. revealed a striking difference of the degree of valley polarization between the exciton and the trion with magnetic field manipulation. The degree is linearly changed as the magnetic field increases from -7 T to +7 T, where the exciton and the trion show different evolution trends. For the exciton, it shows an “X” pattern with opposite helicity excitations, which implies that Zeeman splitting induces an asymmetry in the intervalley scattering. Exchange interactions of the electron and hole give rise to the difference in excitonic dispersion. While for the trion, it shows a “V” pattern presenting that the degree increases for either sign of magnetic fields [161].

The dark exciton is mysterious and usually vanishes in spectral measurements, because the spin of the electron or hole is opposite resulting in spin-forbidden transitions. By applying an in-plane magnetic field, the magnetic perturbation could brighten the dark exciton. Two PL peaks are observed using a 30 T magnetic field at 4 K, which are determined and verified as a dark exciton and a dark trion by theory calculations. Interestingly, it shows an opposite handedness at the energies of the exciton and the trion, but the precise mechanism behind these observations remains unclear [162]. Besides, some other works study the excitonic diamagnetic shifts under a large magnetic field, which extends the understanding of the Zeeman effect in 2D materials [49, 50].  Figure 5(e) shows the energy change of Zeeman splitting when the magnetic field increases from zero to a high magnetic field (30 T), where the changing trend of energy is similar for various excitons [163].

Ultrafast measurements provide a platform to generate a pseudomagnetic field for the dynamics study of valley polarized excitons.  Figure 5(f) shows that a valley-selective optical Stark effect of the WSe_2_ monolayer can be generated at a nonresonant *σ*+ pump excitation (1.53 eV, below the direct band gap). As a result, the energy levels of the K valley and ground state shift up and down, respectively, but the energy level of the K′ valley remains unchanged. A probe laser with *σ*+ and *σ*- polarized states is used to detect the transient reflection spectra, and the probe energies are controlled from 1.59 eV to 1.77 eV. From the contrast map of reflectivity (Δ*R*/*R*), strong changes are observed only for the *σ*+ probe pulse, and no signal is presented for the *σ*- probe pulse. That means that the nonresonant *σ*+ pump only modifies the optical transitions at the K valley, but not at the K′ valley. Such coherent manipulations of valley polarization with ultrafast measurements open up a convenient way to realize spintronic applications [164].

Moreover, the difference of valley polarization PL can be understood within the dynamic processes using the time-resolved PL measurements. One work reported the valley dynamics of the exciton, trion, and localized state in WSe_2_, where the decay of trion polarization shows a partial and fast decay within 10 ps before reaching a stable polarization of about 20% [165]. Interestingly, Li et al. demonstrated the direct measurements of exciton valley coherence of monolayer WSe_2_ using polarization-resolved optical coherent spectroscopy. The time of exciton valley coherence is determined to be ~100 fs, which is faster than the exciton population recombination < 1 ps [166]. Some work focused on the dynamics of valley polarization in 2D heterostructures. Xu et al. reported that the interlayer exciton of the MoSe_2_/WSe_2_ heterostructure owns a long-lived polarization of about 40 ns. From the spatial maps in Figure 5(g), the difference of exciton PL intensity and distribution demonstrates the valley effect in TMD heterostructures [167]. Kim et al. observed a valley-polarized hole population lifetime of 1 *μ*s and an ultralong valley lifetime up to 40 *μ*s in WSe_2_/MoS_2_ heterostructures [168].

## 5. Heterostructure Based on 2D Materials

Plasmonic nanostructures or optical cavity systems can harvest photons creating local electromagnetic confinements, which help to overcome the bottleneck of limited light-matter interactions in 2D materials. The proposed plasmonic metasurfaces and optical crystal cavities for tailoring valley polariton behaviors are discussed here. They can effectively enhance the valley photoluminescence and also manipulate the exciton emission or the near-field propagation direction of valley excitons. The mechanism of valley exciton detuning can be summarized as two reasons: one is near-field effect induced plasmon-exciton coupling and the other is strong coupling induced photon-exciton polariton.

### 5.1. Plasmonic Nanostructure-Coupled 2D Materials

#### 5.1.1. Manipulation of Exciton Emission

**Figure 6:**
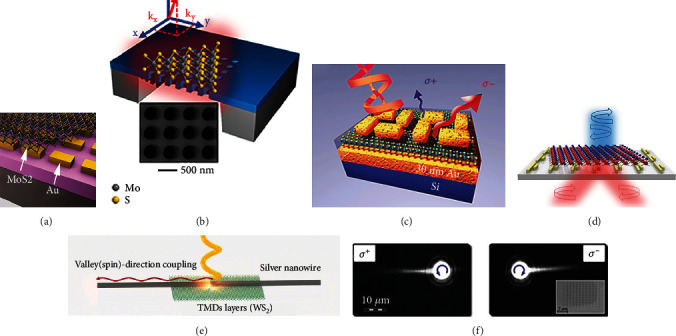
Polarization-dependent optical response in plasmonic nanostructure-coupled 2D materials. (a) Metallic nanorod-induced plasmon-exciton coupling and spectral modification. (b) Giant PL enhancement in photonic nanohole-coupled MoS_2_ monolayer. (c) Hybrid metasurface-coupled MoS_2_ monolayer for valley PL tailoring. (d) Valley-selective directional PL emission of 2D materials separated by metasurface. (e) Directional propagation of valley-coupled surface plasmon polariton in silver nanowire-coupled WS_2_ monolayer. (f) Chiral coupling of valley exciton with optical spin-orbital coupling effect. Panel (a) is reproduced with permission from Ref. [170], copyright 2014 *ACS Nano*. Panel (b) is reproduced with permission from Ref. [171], copyright 2017 *Nano Letters*. Panel (c) is reproduced with permission from Ref. [61], copyright 2018 *Advanced Materials*. Panel (d) is reproduced with permission from Ref. [175], copyright 2018 *Applied Science*. Panel (e) is reproduced with permission from Ref. [64], copyright 2018 *Science*. Panel (f) is reproduced with permission from Ref. [66], copyright 2018 *ACS Photonics*.

The resonance of surface plasmons shows sensitive responses to the structural geometry. Several works reported that hybrid structures can be realized by transferring CVD-grown TMD onto plasmonic nanostructures [169]. In Figure 6(a), the PL intensity of the hybrid MoS_2_-Au nanorod structure is increased up to 65% compared to the bare MoS_2_, which arises from the interactions between nanoantenna and surface plasmon [170]. And the large length-width ratio of the nanorod introduces an optical anisotropy that can be exploited for polarization selective enhancement of light signals, involving scattering, absorption, and PL. The array of the bowtie silver nanostructure can support narrow plasmon-lattice resonances, which has been designed to couple the MoS_2_ monolayer. Benefitting from the stronger coupling of the exciton-plasmon at resonant polarization directions, the PL and Raman intensity show profound enhancements, and even more, the reflection spectrum shows a Fano line shape stemming from the interference between the exciton and the plasmon [60]. Usually, the PL enhancement from the plasmonic nanostructure-coupled TMD monolayer is 100-fold to 1000-fold.  Figure 6(b) reports a 1300-times enhancement in PL emission from a MoS_2_ monolayer via simultaneous Fano resonance induced by a dielectric photonic crystal [171]. Impressively, a recent work demonstrates that a 20000-fold enhancement is achieved by integrating the WSe_2_ monolayer onto plasmonic trenches in a gold substrate, where the gap plasmon modes can be well confined in the trenches [172]. It is found that the largest PL intensity can be observed at 45° polarization excitation. In these works, the electromagnetic field resonance plays the dominate role in polarization-sensitive PL enhancement.

Plasmonic metasurfaces are artificial nanoscale structures with unique distributions and geometries, which can be precisely designed to obtain negative permittivity material for phase control of light [173]. A chiral plasmonic metasurface is designed and fabricated onto the multilayer substrate to realize the manipulation of MoS_2_ PL spectra of distinct valleys, as shown in Figure 6(c). The generation and radiation processes of valley exciton are accompanied by the coupling of a super chiral electromagnetic field, resulting in enhanced light absorption and tailored valley PL. Because of the super chiral field, the value of degree of valley polarization of the MoS_2_ metasurface is increased from 25% to 43% at the *σ*- excitation, while it decreases from 25% to 20% at the *σ*+ excitation. It provides a viable way in the configuration of nanostructure-coupled TMD for manipulating the valley degree of freedom in specific valleys [61].

The change of PL intensity, spectra shape, and emission angle could also be detuned by the coupling of metasurfaces [174]. In a simulation work, excitons in different valleys are regarded as point-dipole emitters with inverse chirality. Double-bar plasmonic nanostructures lead the emission from different valleys into opposite directions, as shown in Figure 6(d), which arises from the interference effect between the dipole and quadrupole modes excited in the neighboring bars [175, 176]. Although the reduced directionality happens while considering the averaging effect, the emission angles can be easily tuned by changing the structure parameters and optical polarization states, which could be applicable for enhanced circular dichroism measurements of chiral molecules.

The discussions and comments on these works are mainly focused on how exciton-plasmon coupling happens. Plasmonic nanostructures can couple far-field light to near-field electromagnetic modes, converting spin and angular momentum information into intensity, direction, and phase distributions of electric and magnetic dipoles. And in physical models, the excitons in TMD can be considered as electric dipoles. Both the near-field electric and magnetic dipoles are considered as the radiative sources. They are coherent with each other and propagate to the far-field generating electromagnetic waves. Actually, excitons and plasmons are excited at the same time, and the coupling process cannot be considered separately as time goes by. It will be proper to understand that the coupled new system should be quasiparticles of exciton-plasmon, which possess the features of excitons and plasmons. Moreover, the near-field tailoring effect is reasonable and robust, because all the works analyze the electromagnetic field modes of plasmons and try to illustrate the coupling mechanism in the collective contribution of electric dipole and extra participation of higher-order electromagnetic modes [177].

#### 5.1.2. Separation of Polariton Propagation

Surface plasmon polaritons (SPPs) are the collective oscillations of free electrons in metallic films and nanowires, where electrons perform period oscillations under the excitations, then propagate towards the interface between metal and dielectric materials. When SPPs couple with TMD excitons, they are usually accompanied with space separation of valley excitons or with the transformation of the valley pseudospin to optical angular momentum or include both processes. The optical angular momentum is a robust and controllable degree to transfer optical intrinsic information with one-to-one transformation because of spin-momentum locking [178]. Utilizing this locking effect, directional propagation of exciton-plasmon polaritons has been realized in several works [63, 179–182].

One structure is the single silver nanowire on top of few-layer WS_2_, shown in Figure 6(e), where the evanescent fields of the SPPs possess transverse optical spin angular momentum in the out-plane direction [64]. The coupling between plasmons and excitons follows the law of energy and momentum matching, among circular transition dipoles and plasmonic eigenstates of its local transverse optical spin. When the transverse optical spin is the maximum, the directional propagation of exciton-plasmon polaritons can be observed at room temperature. Notably, the heterostructure of nanowires and TMD is symmetric, but the asymmetric control can be achieved by adjusting the location of focusing laser spot in the short axis direction of nanowires. The results demonstrate that the induction of asymmetric laser excitation can effectively tailor the polariton propagation and the coupling efficiency.

The spatial separation of polaritons in the hybrid structure of the metasurface and TMD has been proposed in Figure 6(f) [66]. The coupling system consists of a mechanically exfoliated WS_2_ monolayer covering plasmonic hole arrays, with a thin dielectric spacer to avoid PL quenching of WS_2_. The chiral metasurface-coupled valley exciton allows the spin-locked spatial separation, even realizing the directional propagation. At the second harmonic resonant energy of the metasurface and A-exciton, the chiral response of valley information shows a higher distinguished signal than that at the pristine resonant energy. Remarkably, the degree of valley contrast is observed to be 40% at room temperature, and this value is quite large due to the strong coupling effect, where the valley relaxation is demonstrated to be outweighed by the faster Rabi energy exchange between the exciton of each valley and the corresponding plasmonic mode.

Another work on the study of valley exciton separation and routing has been achieved in an asymmetric groove metasurface with the MoS_2_ monolayer [183]. The asymmetric groove metasurface consists of a tilted interface in the slit, where two sidewalls couple with the in-plane dipoles of left- or right-handed chirality unequally, resulting in net unidirectional propagation of SPPs with valley excitons. By placing MoS_2_ on top of a metasurface, not only valley excitons are separated and observed in real space, the emitted photons with different helicities can also be separated in momentum space to the far field at different reflection angles. The degree of valley polarization has been verified as an asymmetry PL signal from the MoS_2_ metasurface and a vanishing valley degree from the MoS_2_-flat silver film.

The potential abilities for the separation and the directional propagation of valley excitons have been recognized, which allow further valleytronic and valley photonic applications. This coupling strategy holds the priorities in two parts: one is the exciton Hall effect and the other is the high valley polarization efficiency working at room temperature. The grating or metasurface could couple the angular momentum of valley exciton and convert it to in-plane propagation momentum. This crucial concept extends the photonic spin Hall effect to the function of energy and momentum information delivery. The bottlenecks of the enhancement and control of the degree of valley polarization at room temperature require desirable designs of photonic devices. These photonic devices are based on near-field coupling of SPP propagation and valley excitons, which allow future approaches in controllable exciton-spin-valley transport at room temperature.

### 5.2. Strong Coupling

#### 5.2.1. Strong Coupling in Optical Microcavity

Control of the light-matter interaction is a critical step to develop photonic devices of 2D materials, which may improve their limited absorption and emission efficiency in ultrathin atomic thickness. Optical cavities with a pair of mirror layers provide multilevel reflections and increase the light routes between two reflection interfaces in a microstructure [184]. When the light incidents into a microcavity, photons can be trapped and confined in the cavity. The light in the cavity reflects multiple times producing standing waves for a certain resonance energy. The generated standing wave patterns are called microcavity photonic modes. Most of the existing photonic couplings belong to the weak coupling, where the optical cavities only modify the spontaneous rate of photons or the electronic properties of active media. To realize the further optimization of microcavity quality, the coupling intensity could be strengthened to form hybrid quasiparticles, like strong coupling.

**Figure 7:**
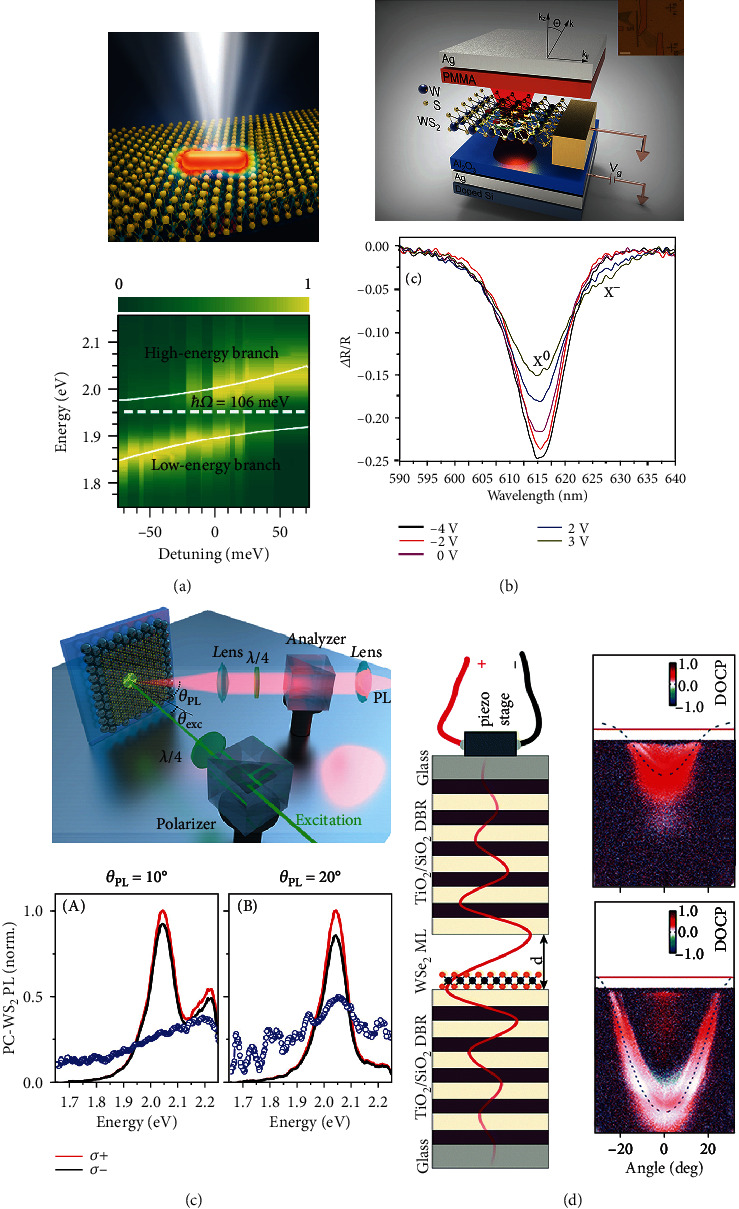
Strong coupling in hybrid structure based on TMD materials. (a) Strong coupling in a hybrid system of MoS_2_ monolayer and golden nanorod. Angle-resolved differential reflection spectra show a Rabbi splitting of 106 meV. (b) Control of strong coupling through electric field gating in a hybrid system of WS_2_ monolayer and Ag reflection layer. The reflection spectra can be detuned by adding gate bias. (c) Strong coupling in a hybrid system of MoSe_2_ monolayer and Tamm-plasmon structure. The valley PL spectra are detected at a low temperature of 5 K. (d) Strong coupling in a hybrid system of MoSe_2_ monolayer and tunable microcavity. The degree of circular polarization (DOCP) can be tailored. Panel (a) is reproduced with permission from Ref. [185], copyright 2017 *Nano Letters*. Panel (b) is reproduced with permission from Ref. [191], copyright 2018 *Nano Letters*. Panel (c) is reproduced with permission from Ref. [198], copyright 2019 *ACS Nano*. Panel (d) is reproduced with permission from Ref. [202], copyright 2019 *Nanoscale*.

Several works reported the strong light-matter interactions in plasmonic nanostructure-coupled 2D atomic crystals. Observed from the scattering spectra of the hybrid systems, they always show two splitting peaks, and the energies of the splitting peaks are sensitive with the detection angles.  Figure 7(a) shows the plasmonic nanorod-coupled WS_2_ flake, where the colored normalized scattering spectra show the detunings between the plasmon resonance and the exciton [185]. This work demonstrates a systematical study in the actively controlled strong coupling process, where the coupling effects on the nanorod size and temperature are discussed. In related works, the effect of strong coupling on TMD layers is studied in the hybrid system of gold bipyramids and multilayer WSe_2_ [186]. And the strong coupling detunings among trions and plasmons have been demonstrated in a hybrid monolayer WS_2_-plasmonic nanoantenna system [187]. Besides, plasmonic lattice modes are promising candidates to show high-quality optical modes and sharp spectral peaks. Several works try to design plasmonic lattices to tailor the strong coupling based on TMD [188, 189]. The angle-resolved differential reflectance spectra can be measured directly to show the detuning processes in various plasmonic lattices. Furthermore, the strong coupling process can be actively detuned by electrical doping [190].  Figure 7(b) shows a device consisting of a WS_2_ field effect transistor embedded inside a microcavity structure. The spectral windows can be detuned by adding gate biases, and the evolution from strong coupling to weak coupling occurs when the monolayer WS_2_ becomes more n-type doping [191].

The strong coupling mechanism in an optical cavity can be described as follows: when TMD flakes are placed in an optical mirror, such as a metallic reflection layer or distributed Bragg reflector (DBR) microcavity, excitons can effectively interact with additional modes of electromagnetic radiations [76]. In such a situation, the radiative photons can be reabsorbed by TMD materials and reemitted again. The emission-absorption process happens repeatedly between excitons and photons, until either the excitons vanish in a nonradiative decay or the photons escape from the oscillation cavity. If the excitation energy is coherently transferred between the exciton and the photon overcoming those above decay processes, the strong coupling regime is created and established. The obvious phenomena observed from energy spectra are that new eigenmodes of system are formed and split with a big Rabi energy. In the framework of strong coupling regime [192], the excitons in matter and the photons in the microcavity can be regarded as two quasiequivalent particles with mutual interference, generating new half-photon-half-matter quasiparticles, namely, polaritons. Polaritons possess intrinsic properties such as low effective mass and strong interaction, which could lead to the experimental realization of a wealth of novel physical phenomena such as Bose–Einstein condensation, superfluids, solitons, and optical spin switch.

#### 5.2.2. Valley Polariton with Strong Coupling

Valley polaritons can be established in a microcavity with embedded TMD flakes in a strong coupling regime [193]. The large binding energy and oscillator strength of TMD excitons help to create the formation of quasiparticles even at room temperature, which allows the detection of valley polarization and reduces the barriers of valley exciton manipulation. Marrying strongly coupled quasiparticle systems with the polarized excitation of valley excitons, polaritons could enable new physical phenomena in nanophotonics with optical spin and material valley [194, 195]. It may open up new avenues for the explorations of spin- and valley-dependent polariton interactions in TMD microcavity systems.

The early observations of strong coupling in experiments are reported in a dielectric bragger reflection (DBR) cavity-coupled MoS_2_ monolayer [196]. Angle-resolved PL spectra show two-branch emissions consisting with the reflectivity results, and a Rabi splitting of 46 ± 3 meV has been obtained. For TE polarized excitation at small angles (≤20°), two prominent modes are observed and identified as the lower polariton branch (LPB) and the upper polariton branch (UPB). While in these initial experiments, the PL intensity is weak, and the anticrossing modes could not be fully mapped out. Another work reports the MoS_2_-coupled DBR microcavity, where both the PL and reflectivity spectra show split peaks (UP and LP peaks) at a range of angles (0°-20°) [65]. The energy of Rabi splitting is obtained as 39 ± 5 meV at 13.5°, and the coupling constant is calculated from a couple oscillator cavity model. The degrees of valley polariton PL are investigated at 8 K, where the degrees are 19% and 29.5% for UP and LP peaks in the MC-MoS_2_ structure, respectively. Although the degree does not show attractive improvements compared with that of bare MoS_2_ as 40%, the superiority of strong coupling effect shows a good preservation of valley polarization at room temperature, as a dramatic increase of degree from 0 (bare MoS_2_) to 13% (MoS_2_-DBR) [197].

Besides, the room-temperature strong coupling can also be realized in a WS_2_-coupled metallic optical cavity. The cavity structure consists of a silver mirror, SiO_2_ dielectric layer, and WS_2_ layer. A series of cavity structures have been fabricated and compared with the detuning energies of −105 meV, −60 meV, and +16 meV, and their corresponding Rabi splitting energies are 100 meV, 80 meV, and 70 meV, respectively. The degree of valley polarization shows an angle-dependent distribution, which is attributed to the increased proportions of the exciton in polariton states at large angles. Recently, the tunable valley polaritons of the WS_2_ monolayer have been reported with the substrate of self-assembled plasmon crystals. A remarkable energy of the Rabi splitting has been recorded as large as 160 meV, which is detected from transmission spectra. The valley-polarized PL and the degree of polarization can be tailored at room temperature, as shown in Figure 7(c) [198]. Another work reported that strong coupling can be established in a Tamm-plasmon structure embedded with the MoSe_2_ monolayer [199]. The engineering of the substrate contributes to the transfer of the oscillator length from exciton to trion energy, resulting in the manifestation of strong coupling by energy-momentum dispersion relation. The valley polarization is macroscopically preserved under the polarized pump, showing a circular polarization degree of 13% and a linear polarization degree of 26%.

Moreover, some special optical cavities show unique photonic confinement and flexible tunability. A recent study on Laguerre-Gaussian cavity-coupled MoSe_2_ presents a 3D confined strong coupling with distinguished excitons and trions [200, 201]. The Rabi splitting energies are simulated as 15.2 ± 0.1 meV for excitons and 1.3 ± 0.1 meV for trions. In the dynamics of strong coupling regime, the valley depolarization can be suppressed, which shows robust valley polaritons with large polarization degrees. Interestingly, the valley polaritons can also be realized in a voltage-tunable dielectric microcavity-coupled WSe_2_, where the strong coupling process can be opened and tuned by a piezo stage, as shown in Figure 7(d) [202]. The degree of valley polarization can be tuned in a wide range. It demonstrates the possibility of manipulating valley polarization PL and suppressing intervalley scattering of excitons in a strong coupling-induced valley polariton system.

In the hybrid system of quasiparticles with strong coupling of valley excitons and cavity photons, the valley degree of freedom with specific angular momentum is induced and manipulated at splitting energies. The hybrid quasiparticle is part of photons and valley excitons, where the proportion of each part is sensitively related with the coupling strength, detection angle, and resonant energy. When the exciton proportion dominates the hybrid system, the strongly coupled quasiparticle shows more valley exciton behaviors and vice versa. The contribution of strong coupling in valley excitons has two sides; on the one hand, the cooperation of photons decreases the degree of valley polarization of polaritons at low temperature, compared to the pure TMD monolayer. On the other hand, benefiting from the strong coupling, the momentum of spin-valley coupling can be reserved even at room temperature.

## 6. Optoelectronic Devices

TMD have been studied and developed to realize various optoelectronic devices, including field-effect transistors, photodetectors, and light-emitting devices [203–205]. In this part, we review the polarization-dependent light-emitting and optoelectronic devices. Some are related with the pristine anisotropic properties of materials, some are attributed from the valley selective PL, and others are based on the coupling of chiral nanostructures. These functional devices have attracted considerable attention in the application of ultrasensitive photodetectors and controllable light-emitting devices. Another important point is that 2D TMD provides abundant physical phenomena and full compatibility with mature semiconductor processing, which could promote the development of mass productions and practical applications in the future.

### 6.1. Light-Emitting Device

**Figure 8:**
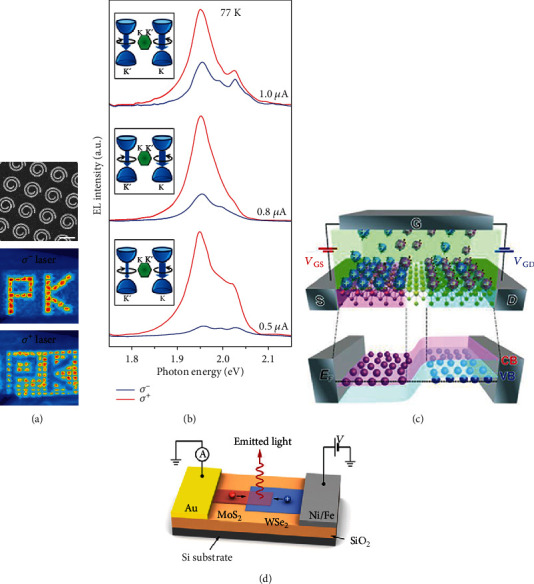
Light-emitting devices based on 2D materials. (a) Chiral spiral nanostructure-coupled MoS_2_ monolayer shining “concave” and “convex” emitting patterns. (b) Electrically controlled valley-light-emitting devices based on the WS_2_ monolayer. (c) Valley polarization light emission from a spin-injected heterojunction. (d) Electrically switchable chiral light-emitting transistor based on the WSe_2_ monolayer. Panel (a) is reproduced with permission from Ref. [209], copyright 2016 *ACS Nano*. Panel (b) is reproduced with permission from Ref. [10], copyright 2016 *Nano Letters*. Panel (c) is reproduced with permission from Ref. [210], copyright 2014 *Science*. Panel (d) is reproduced with permission from Ref. [9], copyright 2016 *Nano Letters*.

The flexible control of light emission of valley excitons draws great attention, which can be manipulated with an alternative degree of freedom of light polarization [206–208]. By fabricating hybrid structures with subwavelength Au spiral rings and a MoS_2_ monolayer, the active manipulation of MoS_2_ PL intensity can be realized. Due to the exciton-plasmon interaction induced by optical spin-orbit coupling (SOC), PL intensity is dramatically enhanced and reversibly controlled by changing the spin state of photons, the laser power, and the structure geometry. Furthermore, the spiral rings with clockwise and anticlockwise rotations show chiral geometric properties, which can be designed as emitting units to construct multipattern light-emitting devices. 2D light-emitting devices based on this SOC effect are successfully achieved as the shining pattern “PK” shown in Figure 8(a) [209].

A valley-light-emitting diode (VLED) can be realized with electrical excitation by injecting electrons and holes in the TMD monolayer. The circularly polarized electroluminescence (EL) of the WS_2_ monolayer has been measured in a p-i-n heterojunction (*p*-Si/*i*-WS_2_/*n*-ITO). Under forward bias, electrons and holes are injected from the n-ITO and p-Si layers, respectively. The recombination of electrons and holes results in radiative luminescence peaks around 2.0 eV, involving *X*^*b*^, *X*^0^, and *X*^−^ exciton peaks. At 77 K, the polarized EL of the device has been detected, showing that the *σ*+ component of EL is stronger than the *σ*- component, as shown in Figure 8(b). The degree of circular polarization of the total EL shows a value as large as 81% at 0.5 *μ*A and gradually retrogrades to 20% as bias voltage increases [10].

However, the modulation of the amplitude of degree of valley polarization in electronic devices is not enough. Due to the development of practical applications, there is a strong demand for the realization of a circularly polarized light source with fully electrical control. Iwasa et al. reported that WSe_2_-based ambipolar transistors can emit circularly polarized light at a p-i-n junction, as shown in Figure 8(c), which can be further switched between inverse degrees of valley polarization by controlling the in-plane electric field [210]. By changing the direction of the source and the drain, the emission energy can be tuned due to the doping-induced competition of excitons and trions. The phenomenon has been explained in the mechanism of electric field-induced change of charge overlap and transition possibility.

Besides, the investigations of spin carrier injection and manipulation of TMD electronic devices have been explored to integrate with the magnetic field and material. Zhang et al. reported the electrical generation and the control of spin charges from the ferromagnetic semiconductor to the WS_2_ monolayer. WS_2_ monolayers are exfoliated on the (Ga, Mn)As substrate keeping a clean interface for effective spin injection. When a forward bias is applied on the substrate, the spin holes are injected into the WS_2_ monolayer giving rise to the radiative recombination with unpolarized electrons. The magnetic field direction controls the spin state of injecting holes resulting in the selective enhancement of specific polarized emissions [46].

Another way to manipulate the spin charge injection of 2D TMD is choosing ferromagnetic electrodes. As reported in a p-n heterojunction device based on WSe_2_ and MoS_2_, lateral transport of spin-polarized holes has been realized in the WSe_2_ layer by using a permalloy Ni/Fe electrode, as shown in Figure 8(d). Under the positive bias voltage to the permalloy electrode, holes and electrons are generated from Ni/Fe and Au electrodes, respectively, and then meet at the overlapped region resulting in electroluminescence [9]. The direction and the intensity of the magnetic field control the spin-hole injection in the WSe_2_ monolayer, and the degree of valley polarization can be manipulated by the recombination efficiency of electrons and spin holes, stemming from imbalanced charge distributions in two valleys. The demonstration of spin injection and magnetoelectronic control over valley polarization allows a host of novel valleytronic devices based on TMD semiconductors.

### 6.2. Photodetection of Anisotropic Materials

**Figure 9:**
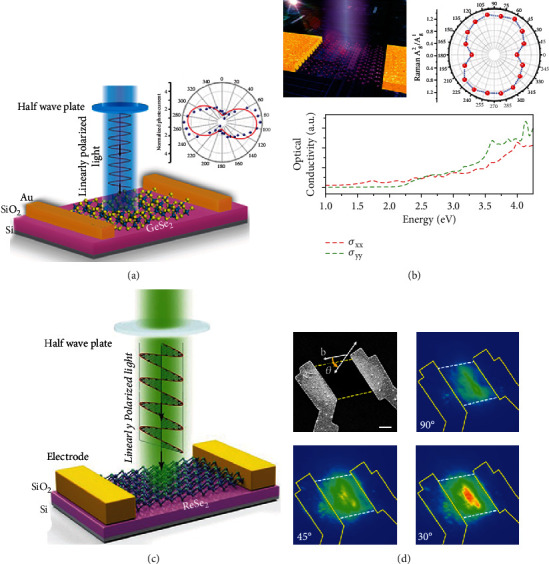
Photodetectors and photocurrent mapping based on anisotropic 2D materials. (a) Schematic view of ultrasensitive photodetector based on 2D GeSe_2_. (b) Ultraviolet photodetector and anisotropic photoresponsivity of few-layer BP. (c) Schematic view of highly sensitive photodetector based on anisotropic ReSe_2_ nanosheets. (d) Polarization-sensitive ReSe_2_ photodetection device and its photocurrent mapping at various polarization directions of incident light. Panel (a) is reproduced with permission from Ref. [212], copyright 2018 *Journal of the American Chemical Society*. Panel (b) is reproduced with permission from Ref. [214], copyright 2015 *ACS Nano*. Panel (c) is reproduced with permission from Ref. [125], copyright 2016 *Advanced Functional Materials*. Panel (d) is reproduced with permission from Ref. [57], copyright 2016 *ACS Nano*.

Recently, the main challenges of photodetection based on 2D materials are extended from the high responsivity covering a broad spectral range to the high sensitivity of polarization. 2D anisotropic materials have unique atomic structures and intrinsic energy bands, which satisfy the requirement of ultrasensitive detection in a wide range of electromagnetic spectra. The wide bandgap photodetectors based on 2D materials have been rarely reported. 2D GeSe_2_ and GeS_2_ own wide bandgaps about 3 eV exhibiting highly polarization-sensitive photoresponses due to the optical absorption anisotropy [211].  Figure 9(a) shows the GeSe_2_ photodetector and its anisotropic photocurrent, where a high dichroic ratio of 3.4 is characterized at 450 nm. The photoresponse is dominant at 300 nm, sharply decreases at the wavelength of approximately 400 nm, and vanishes at zero near 500 nm [212]. The widest bandgap of 2D anisotropic semiconductors should be GeS_2_, which is introduced as an ideal candidate for polarization detection in ultraviolet range [213].

BP flakes have anisotropic optical responses and tunable direct band gaps, which are ideal materials meeting the requirement of infrared photodetection with high sensitivity and fast response.  Figure 9(b) shows a few-layer BP photodetector working in a wide spectrum ranging from near-ultraviolet (UV) to near-infrared (NIR) [214]. The photocurrent diagram shows a high anisotropy along two crystal axes. Another work prepared a BP vertical p-n junction to realize the separation of photoinduced electrons and holes in the device channel [17]. A ring-shaped Au electrode is designed as an anisotropic photocurrent collector to exclude the influence of electrode geometry. The magnitude of photoresponsivity at 1200 nm is measured as large as 0.35 mA W^−1^ with a large contrast ratio of 3.5. Besides that, a photodetector based on 4.5 nm BP flakes has been demonstrated to show a sensitive and high photoresponse in the ultraviolet spectral range, showing a detectivity of 9 × 10 [13] Jones and a responsivity of 9 × 10^4^ A W^−1^ at the voltage of 3 V. One work records the high-contrast photocurrent patterns detected from the BP photodetector on Si/SiO_2_ substrates, in both of the visible (532 nm) and infrared (1550 nm) spectral ranges. The performance of BP optoelectronic devices can be improved by selecting the metal contact. The BP device with a Ni/Au electrode shows an ultrahigh photoresponsivity up to 10^6^ A W^−1^ from 400 nm to 900 nm. Similar to BP, 2D GeAs_2_ and GaSe_2_ semiconductors hold low symmetry structures to present highly anisotropic photodetectivity with a linearly dichroic ratio of 2-4 [95, 211].

The electronic transport property has a pronounced effect on the performance of photodetection. In Figure 9(c), Liu et al. reported that the electron mobility of the ReS_2_ transistor is detected as 40 cm^2^ V^−1^ s^−1^, which contributes to a large on/off ratio of 10^5^ and a good photoresponse of 10^3^ A W^−1^ [125]. The magnitude of photocurrent with the polarized excitation along the *b*-axis shows much greater signals than that along the *a*-axis, which consists well with the anisotropic properties of light absorption. The design of defect engineering of 2D materials is a useful method to improve the electron mobility. O_2_ plasma treatment is induced to create defects in ReS_2_ films, which can affect the drain current at the off state and the recombination of carriers. The mobility of the ReS_2_ film is detected as high as 7.6 cm^2^ V^−1^ s^−1^, which helps the device to get a high on/off ratio of 10^4^ and a high photoresponsivity of 10^7^ A W^−1^, as well as a fast temporal response (rise time of 670 ms) [215].

As the twin sister of ReS_2_, ReSe_2_ has been predicted to exhibit excellent photoresponses.  Figure 9(d) shows the successful preparation of few-layer ReSe_2_ photodetectors on h-BN substrates; the carrier mobility of a back-gate FET device has been significantly enhanced over 500 times for electrons and over 100 times for holes at low temperature, which is due to the dangling bonds and charge impurities on atomically flat surfaces. Based on polarization-dependent photocurrent mapping, the ReSe_2_ photodetectors are determined to hold a high-speed photoresponse with the time down to 2 ms. The device enables a gate-tunable photoresponse in the regime of both the electron and hole contribution in an ambipolar device [57]. Zhai et al. reported anisotropic photodetectors based on alloy ReS_2*x*_Se_2(1−*x*)_ monolayers, whose band gap can be modulated in the range of 1.3-1.6 eV. The anisotropic photocurrent distribution can also be observed in these alloy monolayers, where the maximum and minimum values are detected when the incident light polarizes parallel and perpendicularly to the *b*-axis, respectively [101, 216].

### 6.3. Spin-Valley Photocurrent Detection

In the 2D electronic system (2DES) with spin degeneracy lifted, inhomogeneous distribution of photoinduced carriers can be formed in *k*-space following optical selection rule and energy/momentum matching. Such carriers finally contribute to the generation of the spin current, which has a sensitive fingerprint information with the polarized state of excitation light, namely, circular photogalvanic effect (CPGE) [217, 218]. Generally, in a 2D electronic system with Rashba spin splitting in band structure, such as quantum well and 2D TMD semiconductors, the amplitude of CPGE photocurrent can be expressed as *j* = *ηγI*sin*θ*sin2*φ*, where *η* is the absorption efficiency; *γ* is the matrix element referred to spin, orbital, and symmetry information; *I* is the incident light intensity; and *θ* and *φ* are the incident angle and rotation angle difference between the linear polarizer and quarter waveplate, respectively. Usually, the periodic current intensity can be detected by changing *φ* at a fixed angle of *θ*, and the distribution of periodic current can be fitted well by tuning *γ*.

Cui et al. earlier reported the detection of CPGE current in electrical double-layer transistors based on WSe_2_. The incident-angle-dependent photocurrent measurements are performed at the center position of transistors. The maximum intensity of the photocurrent is recorded at the incident angle of 60°, which is quite similar to the CPGE current observed in Rashba 2DES. At the nonzero incident angle, the detected photocurrent shows a strong dependence on the light polarization, which destabilizes up and down with the rotation angle of the quarter-wave plate. The magnitude of the photocurrent can be manipulated by changing the gate voltage, which can be realized to a level above two orders larger than that of the zero bias case. From the periodic distribution of the photocurrent under different incident angles, the physical mechanism can be depicted in that helicity-dependent photocurrent arises from the asymmetric optical excitation of splitting bands [219].

**Figure 10:**
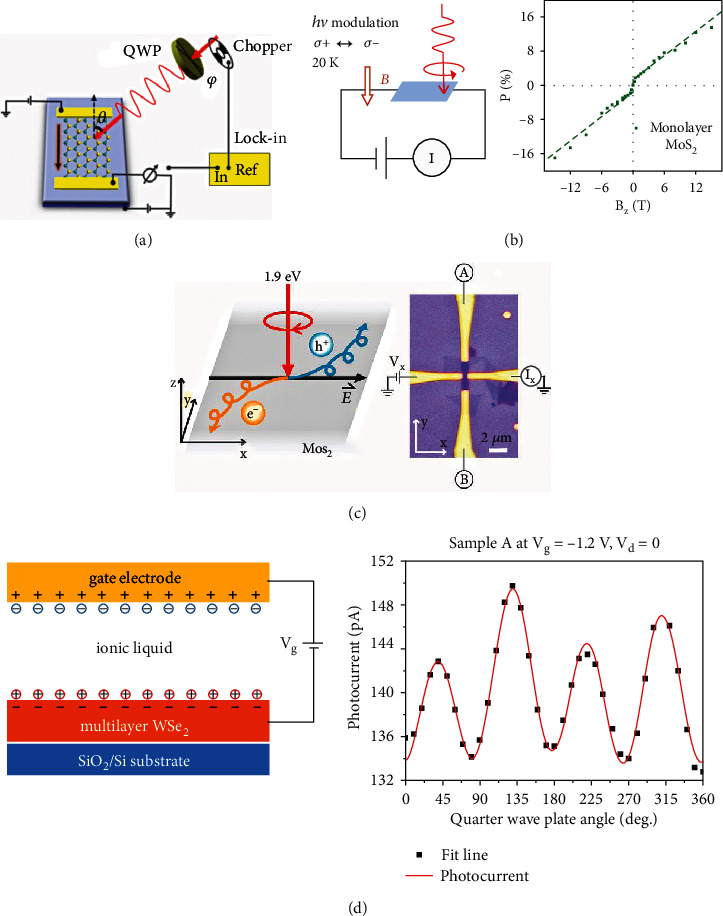
Spin-valley photocurrent detection in 2D materials. (a) Schematic view of spin-valley-coupled CPGE detection. (b) Zeeman effect induced valley photocurrent detection under magnetic field. (c) Schematic view of valley Hall effect and its transistor device based on MoS_2_. (e) Valley Hall effect in multilayer WSe_2_ and its CPGE photocurrent. Panel (a) is reproduced with permission from Ref. [222], copyright 2018 *ACS Applied Materials & Interfaces*. Panel (b) is reproduced with permission from Ref. [223], copyright 2019 *Physical Review Letters*. Panel (c) is reproduced with permission from Ref. [47], copyright 2014 *Science*. Panel (d) is reproduced with permission from Ref. [228], copyright 2019 *ACS Nano*.

A few works demonstrate the electrical control of CPGE in 2D semiconductors [220, 221]. Both of the magnitude and the polarization degree of the photocurrent can be tuned actively, which arises from the spin-valley coupling induced photogenerated carriers. The amplified photocurrents can be modulated up to 45 times, and the polarization degree of the total photocurrent can be tuned from 0.5% to 16.6% significantly, as shown in Figure 10(a) [222]. Moreover, the symmetry of the photocurrent has been systematically studied that it can be modulated by the excitation wavelength, the drain-source voltage, and the azimuthal and the incident angle.  Figure 10(b) shows the schematic view of valley-sensitive photocurrent detection, where the variation of the photocurrent exhibits a comparable quasilinear increase with increasing magnetic field. This result reveals the intrinsic response of TMD under the out-of-plane magnetic field, which is related to the Zeeman effect [223].

As we know about the spin Hall effect, spin carriers with opposite spin states in a current can shift towards to inverse directions with the help of magnetic field [224, 225]. In TMD monolayer, the broken inversion symmetry performs as an effective magnetic field, which not only determines the valley optical selection rules but also creates charge carriers with anomalous velocities. In a valley Hall device, electrons from inequivalent valleys experience opposite Lorentz-like forces resulting in opposite directional movements perpendicularly to the drift current [226, 227].  Figure 10(c) shows that the circularly polarized light is chosen to excite the Hall bar device, where the broken time-reversal symmetry generates a normal drift current and a net transverse Hall voltage. The studies of photoinduced anomalous Hall voltage and resistance provide the strong experimental evidences. A small but finite Hall voltage *V*_*H*_ changes linearly with *V*_*X*_ during the polarization modulation from right- to left-hand circular polarization (R-L), which is the signature of photoinduced anomalous Hall effect driven by a net valley polarization. The sign of *V*_*H*_ flips to the opposite signal when the excitation is changed to L-R modulation [47].

The observation of valley Hall effect in bilayer MoS_2_ faces a great challenge that the material is a centrosymmetric crystal, where the Berry curvature and the valley Hall effect vanishes. Shan et al. reported the breakthrough that the broken symmetry of bilayer MoS_2_ transistors can be induced by using gate voltages. A perpendicular electric field can be applied in the MoS_2_ bilayer to generate a few meV potential differences between two layers [46]. Another work reports the inversion-symmetry-breaking induced valley Hall effect in the WSe_2_ multilayer, as shown in Figure 10(d). The charge doping can be generated in an ionic liquid device, and it can be seen that the amplitude of the CPGE current becomes larger with the increase of gate voltages, demonstrating that the spin-orbit coupling gets stronger at a high doping level [228]. The modulation of the electric field shows a significant effect on the Berry curvature at K and K′ points [44]. The explorations of valleytronic manipulation of 2D semiconductors hold great promises in the development of new-type carrier devices [40, 229].

### 6.4. Photodetection of Heterostructures

**Figure 11:**
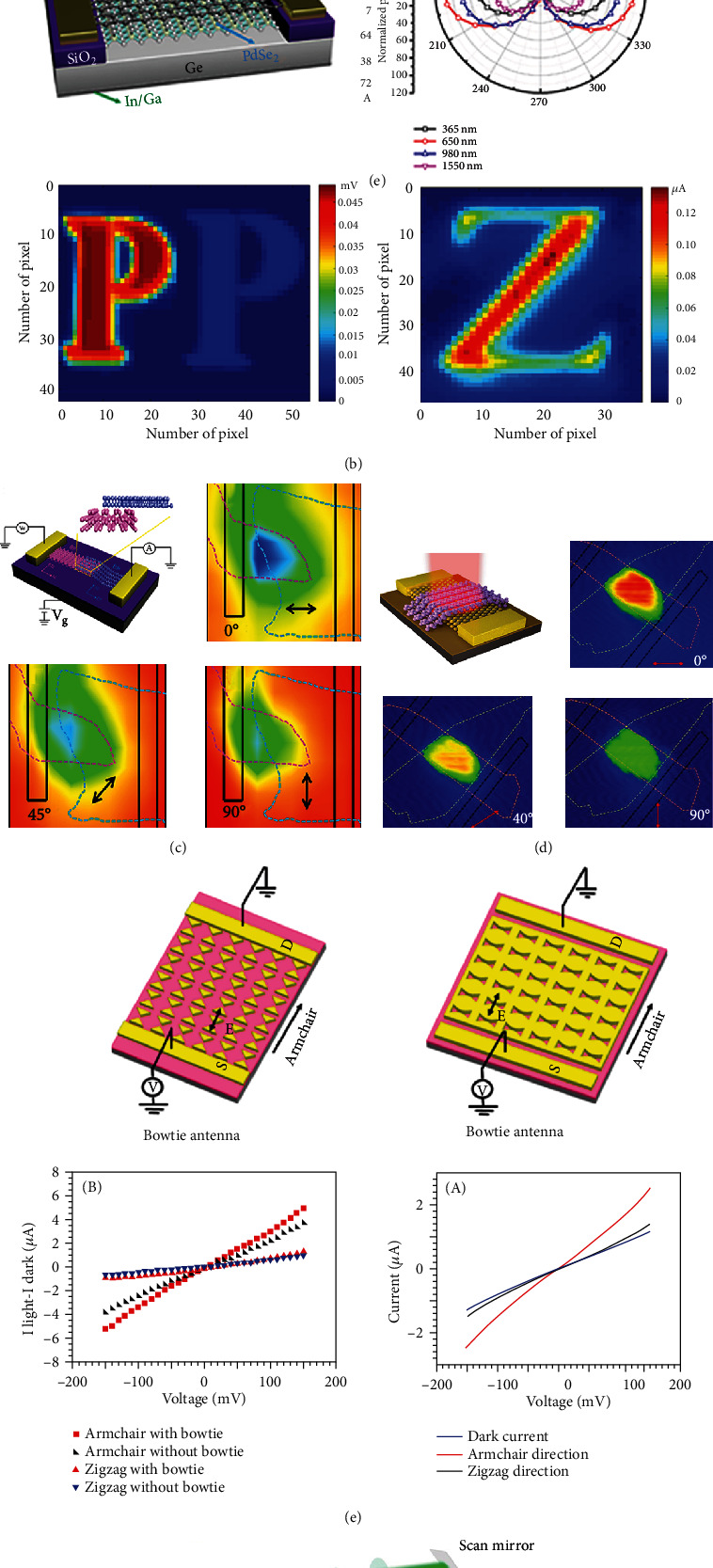
Photodetectors based on 2D heterostructures. (a) Optical image and angle-resolved photocurrent mapping of BP/InSe photodetector. (b) Self-powered photodetector and its polarization imaging based on graphene/PdSe_2_/Ge heterojunction. (c) Polarization-sensitive photocurrent mapping of BP/BP van der Waals junction. (d) Highly polarization-sensitive photodetector and its photocurrent mapping based on BP/WSe_2_ vertical heterostructure. (e) Plasmonic nanostructure-coupled BP photodetectors and their enhanced polarization-sensitivity photoresponses. (f) Plasmonic hot electrons induced photocurrent response of MoS_2_ monolayer with metal gratings. Panel (a) is reproduced with permission from Ref. [241], copyright 2018 *Advanced Functional Materials*. Panel (b) is reproduced with permission from Ref. [243], copyright 2019 *ACS Nano*. Panel (c) is reproduced with permission from Ref. [246], copyright 2018 *ACS Applied Materials & Interfaces*. Panel (d) is reproduced with permission from Ref. [240], copyright 2017 *Nano Energy*. Panel (e) is reproduced with permission from Ref. [250], copyright 2018 *ACS Nano*. Panel (f) is reproduced with permission from Ref. [251], copyright 2015 *ACS Nano*.

The construction of 2D heterostructures provides potential choices to build various types of band alignments [144, 230–232], which help to improve devices' optoelectronic properties due to the enhanced light absorption, the induced built-in electric field, and the improved device resistance [233–236]. Particularly, in the heterostructure system involving isotropic and anisotropic 2D materials, the unique band alignment and the mixed-dimensional exciton absorption would strongly manipulate polarization-dependent optical properties for the design of high-performance photodetectors. Several works report the construction of heterostructures with the linear dichroism of p-type BP flakes and other n-type 2D semiconductors, and they show advanced photoresponses and polarization-sensitive detections [237–240]. The device based on BP-InSe vertical p-n heterojunction demonstrates a wide-spectrum photoresponse ranging from 400 nm to 950 nm, and the anisotropic photocurrent mapping is shown in Figure 11(a) with an anisotropic ratio of 0.83 [241].

The studies of BP-MoS_2_ photodetectors have been extensively reported. In 2014, a photodetector with few layers of BP (11 nm) and the MoS_2_ monolayer is earlier demonstrated to show good current-rectifying effect and high photocurrent responsivity [238]. In 2015, a MoS_2_-BP heterostructure device reports a competition effect between MoS_2_ and BP in the junction region by selecting the excitation energy of lasers [237]. When the incident photon energy is below the band gap of MoS_2_ but above the band gap of BP, the photocurrent distribution of the device is similar to that of the pure BP device, showing a sensitive detection of light polarization. One work demonstrates a BP/MoS_2_ heterostructure to fabricate photodiodes, which can be operated to realize the detection of light intensity and polarization in microwave infrared (MWIR) range at room temperature. The device exhibits remarkable optoelectronic performance comparable to conventional MWIR photodetectors, where the quantum efficiency and specific detectivity are recorded as high as 35% and 1.1 × 10^10^ cm Hz^1/2^ W^−1^, respectively. It is the first device to show polarization-resolved photoresponse under the control of gate bias [242].

Figure 11(b)shows the self-powered photodetector based on the graphene/PdSe_2_/germanium heterostructure, which shows a highly polarization-sensitive photoresponse in a broadband spectra ranging from deep ultraviolet to midinfrared [243]. Owing to the enhanced light absorption in mixed-dimensional heterojunction and fast carrier transport, the polarization sensitivity is recorded as high as 112.2. By utilizing the metal mask controlled by 2D motorized stage, the device can be further applied to show polarization-dependent photocurrent mapping with a polarization contrast ratio over 10. Another work reports a broadband photodetector of 2D PdSe_2_/MoS_2_ heterostructure, which demonstrates excellent air stability over three months with a high responsivity of 185.6 mA/W [244]. To overcome the limitation of optical absorption, a PdSe_2_/Si mixed-dimensional heterostructure device is reported to show a good responsivity up to 300 mA/W and an impressive detectivity of 1.18 × 10^13^ Jones [245].

The interface of heterostructure plays a significant role in influencing the performance of optoelectronic devices.  Figure 11(c) shows the device of orientation-perpendicular bilayer BP junction and its angle-dependent photocurrent mapping [246]. By constructing the BP homojunction involving armchair and zigzag directions, the device shows localized and remarkable polarization-sensitive photoresponse within a unique rectification mechanism. The main region of photogenerated current can be tailored as the polarization angle of excitation laser rotations. In a BP-WSe_2_ vertical heterostructure device of Figure 11(d), the photogating effect is induced to establish the highly polarization-sensitive infrared photodetector. In this device, WSe_2_ serves as the conductive channel and BP without the contacted electrode works as the photogate. The ultrahigh photoresponse reaches up to ~10^3^ A W^−1^ in the visible range, as well as ~5 × 10^3^ A W^−1^ in the infrared range. Scanning photocurrent microscopy images exhibit spatially dependent anisotropic photoresponses at 1550 nm. And the anisotropic ratio of the photocurrent is observed at about 6 for infrared detection at room temperature [240].

Besides that, the MoS_2_/GaAs n-n heterojunction shows a wide photoresponse range from deep ultraviolet to near-infrared. This self-powered photodetector demonstrates a high polarization sensitivity with a peak-to-valley ratio of 4.8 [247]. The mixed-dimensionality TiS_3_/Si p-n junction presents a wide response spectrum from 405 to 1050 nm and a high anisotropic photocurrent ratio of 3.5 [248]. Notably, the photoresponsivity has been reported to be enhanced in isotropic/anisotropic heterostructure compared to its monolayer, such as MoS_2_/BP, WS_2_/BP, SnSe_2_/MoS_2_, GeSe/MoS_2_, and ReS_2_/MoS_2_. The type of band alignment in heterostructures is quite important in deciding the carrier transfer. In the system of isotropic/anisotropic heterostructure, the type-I heterostructure holds the potential to strongly manipulate polarization-dependent optical properties and optoelectronic devices, because they provide the efficient recombination of spatially confined electrons and holes.

To enhance the intrinsic polarization sensitivity of photodetectors based on 2D materials, plasmonic nanostructures can be designed to enhance the light harvesting and gain the photoresponsivity [249].  Figure 11(e) shows the schematic view of the heterostructure device based on bowtie-structure-coupled BP. With the coupling of bowtie antenna, the device can obviously enhance the optical absorption along the armchair direction by localized surface plasmon resonance, which also results in increased photocurrents. For a bowtie-aperture-coupled BP device, the device demonstrates a high tphotocurrent ratio of 8.7 because the inherent polarization selectivity of BP can be enhanced in near infrared [250]. In Figure 11(f), MoS_2_-metal detectors have been investigated that the maximum photocurrent response occurs at the situation when the light polarization direction is parallel to the metal electrode edge, which can be attributed to the plasmonic hot electron enhanced photovoltaic effect. Normalized photocurrent intensities are plotted and analyzed as a function of incident light polarization with the illumination wavelength ranging from 500 nm to 1050 nm, and the anisotropic ratio of the photocurrent response achieves its maximum around 850 nm [251].

## 7. Outlook and Summary

**Figure 12:**
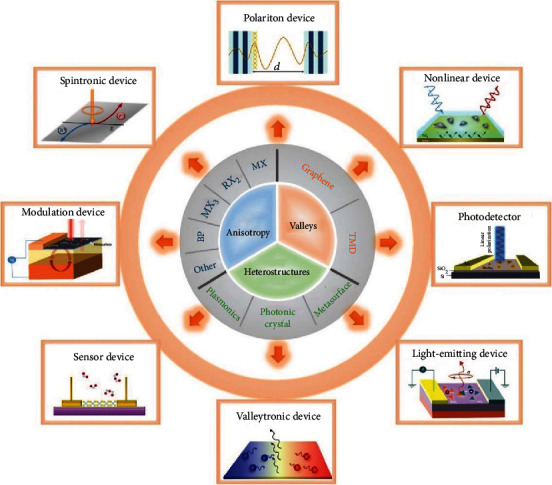
The classification of 2D materials and devices. The polarization-dependent optical mechanism and potential applications in valleytronic, light-emitting, photodetector, nonlinear, polariton, spintronic, modulation, and sensor devices.

Figure 12 classifies the polarization-dependent 2D materials into three types, involving anisotropic 2D materials, 2D Dirac materials with inequivalent valleys, and nanophotonic structure-coupled materials. The group of anisotropic 2D materials is a big family with abundant material systems, such as BP, MX_2_, and RX_2_. Owing to their anisotropic atomic structures, their energy bands exhibit anisotropy in *k*-space. The generation of photogenerated carriers is decided on the anisotropic photoconductivity along crystal directions. The recombination of electron-hole pairs is greatly related to the energy of band gap, as well as the direction of the dipole. The energy band decides the emission energy, and the dipole decides the polarization of emissive photon. Benefiting from the honeycombed atom arrangements in graphene and TMD family, these kinds of materials possess natural Dirac valleys at K point in the Brillouin zone. Considering the spin of electrons and holes, the neighboring K points in gapped bilayer graphene or TMD monolayer are inequivalent due to the broken time-reversal symmetry. Each valley can only couple left or right circularly polarized photons. This process provides the quantum state storage of photons into spin charges, exhibiting intriguing polarization-dependent optical properties. Moreover, nanophotonic structures are artificial optical units, performing as building blocks for the manipulation of photons in the processes of capture, confinement, and modulation. Based on the spin-orbital coupling effect, plasmonic and metasurface nanostructures can couple far-field polarized in-plane waves creating near-field specific electromagnetic modes. The microcavities constructed by photonic crystals can confine photons at specific wavelengths, which arise from the structure-induced multiple-order reflection. When nanophotonic structures are designed to interact with 2D materials, the near-field modes or confined photons couple with excitons generating multibody problems. This multibody physical mechanism provides available routes to extend the application of functional optoelectronic devices.

The applications of 2D materials based on these polarization-response mechanisms can be explored in a wide range of research fields. First are photonic devices; we review the recent works on polariton photonic devices, which open up a great opportunity to hold a high degree of valley polarization at room temperature. Beyond traditional modulation, the study of nonlinear optical response in 2D anisotropic materials or in nanophotonic structure-coupled 2D materials attracts more eyes of researchers. And on the other side, towards the development of mature Si-based integration, more works try to fill the gap of low-dimension materials and CMOS process integration, which could stimulate the research of 2D materials for the application of modulation devices, especially on the modulation of polarization states. Second are the devices with spin-valley physics. Graphene opens up the gate of device applications in spintronics. Beyond graphene, TMD family extends the investigation from spintronics to valleytronics, including the spin Hall effect, valley Hall effect and CPGE. The device performances have been tailored by electric field, magnetic field, and ultrafast laser-induced pseudomagnetic field. So far, the anisotropic ferromagnetic or paramagnetic materials have been rarely reported. In the near future, 2D materials with ferromagnetic and paramagnetic features could rise up as the famous stars. The last ones are the functional optoelectronic devices, such as transistors, photodetectors, and sensor devices. The basic polarization-dependent characteristics of 2D materials have been extensively and comprehensively studied until now. In the next-generation optoelectronic devices, designs should meet the requirement of the military application and the people's livelihood. The advanced optoelectronic devices with faster response, larger anisotropy, and more accurate sensitivity are highly demanded.

In this review, we demonstrate the optical properties and optoelectronic devices of 2D materials showing polarization-sensitive responses. The response mechanism can be summarized as an anisotropic atom structure-induced anisotropic energy band and Dirac semiconductor-induced inequivalent valleys, as well as the multibody coupling system involving plasmons, photons, excitons, and polaritons. In the view of photoconductivity, the polarization-dependent optical responses can be described in theoretical mechanisms. We first summarize the anisotropic 2D materials, among BP, group-IV monochalcogenides, transition metal dichalcogenides, group-IVB trichalcogenides, and others. Then, the inequivalent valleys in gapped graphene and TMD have been discussed, from honeycomb atomic structures, to the Berry curvatures, ending with the multifield-tailored valley polarization PL. Moreover, in the hybrid system of nanophotonic structure-coupled 2D materials, both moderate and strong coupling processes have been classified to exhibit the flexible manipulation of exciton emission and polariton propagation. Based on these unique physical features, these 2D materials hold great opportunities to realize functional optoelectronic devices, which can be applied in extensive research fields among energy storage, quantum information processing, and multifunctional sensing. The polarization-dependent optoelectronics opens up a future avenue towards the vigorous development of low-dimensional materials science and device integration applications.
